# The chromatin remodeler RSF1 coordinates epigenetic marks for transcriptional repression and DSB repair

**DOI:** 10.1093/nar/gkab1093

**Published:** 2021-11-25

**Authors:** Sunwoo Min, Ho-Soo Lee, Jae-Hoon Ji, Yungyeong Heo, Yonghyeon Kim, Sunyoung Chae, Yong Won Choi, Ho-Chul Kang, Makoto Nakanishi, Hyeseong Cho

**Affiliations:** Department of Biochemistry, Ajou University School of Medicine, Suwon 16499, Korea; Genomic Instability Research Center, Ajou University School of Medicine, Suwon 16499, Korea; Department of Biochemistry, Ajou University School of Medicine, Suwon 16499, Korea; Genomic Instability Research Center, Ajou University School of Medicine, Suwon 16499, Korea; Genomic Instability Research Center, Ajou University School of Medicine, Suwon 16499, Korea; Department of Biochemistry and Structural Biology, The University of Texas Health San Antonio, TX 78229-3000, USA; Department of Biomedical Sciences, the Graduate School of Ajou University, Suwon, Korea; Department of Biomedical Sciences, the Graduate School of Ajou University, Suwon, Korea; Institute of Medical Science, Ajou University School of Medicine, Suwon 16499, Korea; Department of Hematology-Oncology, Ajou University School of Medicine, Suwon, Korea; Department of Physiology, Ajou University School of Medicine, Suwon, Korea; Division of Cancer Cell Biology, The University of Tokyo, Tokyo 108-8639, Japan; Department of Biochemistry, Ajou University School of Medicine, Suwon 16499, Korea; Genomic Instability Research Center, Ajou University School of Medicine, Suwon 16499, Korea

## Abstract

DNA lesions impact on local transcription and the damage-induced transcriptional repression facilitates efficient DNA repair. However, how chromatin dynamics cooperates with these two events remained largely unknown. We here show that histone H2A acetylation at K118 is enriched in transcriptionally active regions. Under DNA damage, the RSF1 chromatin remodeling factor recruits HDAC1 to DSB sites. The RSF1-HDAC1 complex induces the deacetylation of H2A(X)-K118 and its deacetylation is indispensable for the ubiquitination of histone H2A at K119. Accordingly, the acetylation mimetic H2A-K118Q suppressed the H2A-K119ub level, perturbing the transcriptional repression at DNA lesions. Intriguingly, deacetylation of H2AX at K118 also licenses the propagation of γH2AX and recruitment of MDC1. Consequently, the H2AX-K118Q limits DNA repair. Together, the RSF1-HDAC1 complex controls the traffic of the DNA damage response and transcription simultaneously in transcriptionally active chromatins. The interplay between chromatin remodelers and histone modifiers highlights the importance of chromatin versatility in the maintenance of genome integrity.

## INTRODUCTION

In eukaryotes, DNA and histones form nucleosomes, which contribute to preserving genomic integrity (1,2). Histone proteins are decorated by post-translational modifications (3), and this epigenetic information is important for nuclear events including transcription, DNA replication, and DNA repair (4). In damaged chromatin, histone modifications are dynamically altered to facilitate rapid repair of DNA breaks (5–7). Recent studies of the chromatin landscape highlight the importance of chromatin dynamics such as chromosome rearrangement and phase separation for efficient double-strand break (DSB) repair (8–10). Moreover, pre-existing histone modifications before DNA damage influence the DSB repair pathway (8,11,12). Thus, chromatin signature decorated by histone modifications is critical for the DNA damage response (DDR).

Under DNA damage, histone modifications switch the chromatin to an inactive transcription status and rapidly silence transcription proximal to the break site (8,13). Ubiquitination of H2A at lysine 119 (H2A-K119ub) is regulated by ATM kinase at DSB sites (13). H2A-K119ub, the most important marker of transcriptional silencing at DSB sites, is mediated by Polycomb repressive complex 1 (PRC1) (14), while histone H3K27 tri-methylation is regulated by Polycomb repressive complex 2 (PRC2) (15,16). The interdependence between these two modifications for transcriptional repression has long been debated, but recent work showed that H2A-K119ub catalyzed by RING1B tethers PRC1 and PRC2 complexes to repressed loci in genome-wide level (17,18). Under DNA damage, EZH2 is rapidly recruited at DSB sites, but H3K27 tri-methylation is rarely changed (19). Thus, so far, histone H2A-K119 ubiquitination promoted by the ATM-PRC1 axis is the most well-known histone modification associated with the DSB-induced transcriptional silencing (20). In this pathway, ATM kinase phosphorylates transcription elongation factor ENL to promote histone H2A-K119 ubiquitination by BMI1 (21), and this modification spreads transcriptional silencing signals a few kilobases from DSB sites, concomitant with propagation of γH2AX (22). In addition to the ATM-PRC1 axis, H2A-K119 ubiquitination is also regulated by the PARP1-FRRUC (FBXL10-RNF68-RNF2 ubiquitin ligase complex) pathway under DNA damage (23). Thus, H2A-K119 ubiquitination is critical for transcriptional silencing at DSB sites. However, the underlying mechanisms responsible for promoting H2A-K119 ubiquitination in pre-existing chromatin contents, as well as the crosstalk with other histone modifications related to DSB-induced transcriptional silencing, remain unknown.

Chromatin remodelers catalyze broad range of chromatin conformation (24). RSF1 (remodeling and spacing factor1) associates with SNF2H ATPase, forming the RSF complex (25). RSF contributes to nucleosome sliding and regulates transcription on chromatin templates (26,27). RSF1 also plays a key role in the maintenance of chromosome integrity (28,29). In the DDR, RSF1 regulates the ATM-dependent DNA damage signaling pathway and DNA repair through the homologous recombination repair (HRR) and non-homologous end joining (NHEJ) pathways (30,31). In addition, RSF1 directly interacts with ATM kinase and is phosphorylated in response to DNA damage (31). Upon DNA damage, RSF1 makes a cell fate decision by controlling the p53-dependent transcriptome (32). In *in vivo Drosophila* and *Xenopus* models, RSF1 contributes to silent chromatin formation through histone H2Av replacement (33) and it preferentially associates with H2Aub (histone H2A-K119ub) nucleosomes, regulating H2Aub-enriched genes (34). Thus, we hypothesized that RSF1 controls chromatin dynamics and transcription status under DNA damage by interacting with histone modifying enzymes.

Here, we demonstrated that histone H2A-K118 acetylation is enriched in transcriptionally active sites and dynamically changed in response to DNA damage. The RSF1-HDAC1 complex is recruited at DSB sites and promotes the deacetylation of H2A(X)-K118 and subsequent ubiquitination of H2A-K119, silencing the transcription at DSB sites. This chromatin change also allows γH2AX propagation and DSB repair, highlighting dual signals for damage-induced transcriptional repression and DDR signaling.

## MATERIALS AND METHODS

### Cell culture

Human U2OS and HEK293T cells were cultured in Dulbecco's modified Eagle's medium (DMEM) containing 10% fetal bovine serum (FBS). Mouse NIH3T3 cells were cultured in DMEM containing 10% FBS. HeLa H2AX KO cells were cultured in DMEM containing 10% FBS. AsiSI-ER U2OS cells were cultured in DMEM (without sodium pyruvate) containing 10% FBS and puromycin (1 μg/ml) and U2OS 2-6-3 and 2-6-5 cells were cultured in DMEM containing 10% FBS and puromycin (1 μg/ml).

### RNA interference, plasmid transfections and mutagenesis

U2OS cells (U2OS, U2OS 2-6-5 and U2OS 2-6-3) were transfected with 100 nM of siRNA using Lipofectamine RNAiMAX and harvested at 48–72 h after transfection. Plasmid transfections were done by using polyethylenimine (PEI, Polysciences) and harvested at 48 h after transfection. The detailed information of siRNA and plasmids is listed in Supplementary Table S1 and S2, respectively. Site-Directed Mutagenesis (Stratagene) was carried out on SFB-H2A and SFB-H2AX to generate SFB-H2A and H2AX -K118R, -K118Q, -K119R, -K119Q, -2KR (K118R and K119R) and H2AX-S139A mutants. The primers used for mutagenesis are listed in Supplementary Table S3.

### Antibodies

The detailed information of antibodies used in this study is listed in Supplementary Table S4.

### Laser micro-irradiation and immunofluorescence

For laser micro-irradiation, U2OS and HeLa H2AX KO cells were seeded on 35-mm round glass bottom dishes (SPL, Korea) and 5-bromo-2-deoxyuridine (BrdU, final 10 μM) was added to the medium for 30 h prior to micro-irradiation. Cells were incubated in a temperature-controlled chamber (37°C, 5% CO_2_), and DNA damage was induced by laser micro-irradiation using 405 nm laser in A1 confocal microscope (Nikon). For live cell imaging, Images were acquired every 1 s for 10 min. For fixed cell imaging, cells were washed three times with PBS after laser micro-irradiation and fixed with 4% paraformaldehyde followed by incubation with 0.5% Triton X-100 in PBS for cell permeabilization. Cells were blocked for 30 min at room temperature in blocking solution (1% BSA in PBS). Primary antibodies were incubated for overnight at 4°C. Next day, secondary antibodies were incubated for 1 h at room temperature and mounted with VECTASHIELD® with DAPI (Vector Laboratories). At least 10 cells were irradiated in every experiment, and representative data are shown.

### Immunoprecipitation and pull down

Cells were harvested and lysed in NETN buffer (50 mM Tris–HCl, pH 8.0, 150 mM NaCl, 0.5% NP-40 and 5 mM EDTA) with protease and phosphatase inhibitors. Cell lysate was sonicated using EpiShear Probe Sonicator (Active motif) and centrifuged at 13 000 rpm for 15 min at 4°C. For immunoprecipitation, the supernatant was incubated with primary antibody for overnight at 4°C. Next day, the immunoprecipitates were captured by incubation with Protein A Sepharose Fast-Flow (GE Healthcare) for 2 h and the beads were washed four times with NETN buffer. For pull down of SFB-tagged proteins, the supernatant was incubated with Streptavidin Sepharose High Performance affinity resin (GE Healthcare) for 2 h at 4°C and washed four times with NETN buffer. The washed precipitates were boiled with 2× sample buffer and subjected to western blotting.

### Chromatin fractionation and western blotting

For chromatin fractionation, cells were harvested and lysed in NETN buffer without EDTA on ice for 20 min at 4°C. The lysate was centrifuged at 13 000 rpm for 15 min 4°C. The pellet was suspended with nuclease-containing lysis buffer (50 mM Tris–HCl, pH 8.0, 150 mM NaCl, 0.5% NP-40 and nuclease (25–50 U)) and incubated for 20 min in 37°C shaking incubator. The supernatant was collected and processed to western blotting. Samples were boiled with 1× sample buffer and resolved by SDS-PAGE using gradient gel (4–20% acrylamide gel). The separated proteins were transferred onto nitrocellulose membrane (Whatman) and blocked for 1 h with 1% BSA in TBST. The membrane was incubated with indicated primary antibodies for overnight at 4°C and with secondary antibodies (Biorad) for 1 h at room temperature. The immunoblotted proteins were detected with ECL reagents (GE Healthcare).

### Chromatin immunoprecipitation (ChIP)

DSB-inducible cells were treated with 4-OHT or 4-OHT/Shield1 to induce DNA damage and cross-linked with 1% formaldehyde for 20 min at room temperature. Cells were lysed with SDS lysis buffer (1% SDS, 10 mM EDTA, 50 mM Tris, pH 8.1) with protease and phosphatase inhibitor cocktail (Thermo) for 10 min on ice. The lysates were sonicated by Bioruptor (Diagenode) and centrifuged at 13 000 rpm for 15 min. The supernatants were collected and diluted with ChIP dilution buffer (0.01% SDS, 1.1% Triton X-100, 1.2 mM EDTA, 16.7 mM Tris–HCl, pH 8.1, 167 mM NaCl) followed by overnight incubation with primary antibody. Next day, 20 μl of Protein A agarose/salmon sperm DNA (Millipore) was added to each sample and incubated with rotation at 4°C. After incubation, beads were washed with the following washing buffer: low salt immune complex wash buffer (0.1% SDS, 1% Triton X-100, 2 mM EDTA, 20 mM Tris–HCl, pH 8.1, 150 mM NaCl), high salt immune complex wash buffer (0.1% SDS, 1% Triton X-100, 2 mM EDTA, 20 mM Tris–HCl, pH 8.1, 500 mM NaCl), LiCl immune complex wash buffer (0.25 M LiCl, 1% IGEPAL-CA630, 1% deoxycholic acid (sodium salt), 1 mM EDTA, 10 mM Tris, pH 8.1), and TE buffer (10 Mm Tris–HCl, 1mM EDTA, pH 8.0). The immune complex was eluted in elution buffer (1% SDS, 0.1 M NaHCO_3_) for 30 min at RT. The eluates were collected and incubated with 10 μl of 5M NaCl to reverse histone-DNA crosslinks at 65°C for 4 h, followed by incubation with proteinase K at 45°C for 1 hr to remove proteins. The remaining DNA was purified with Nucleospin PCR clean up kit (Macherey-Nagel) and processed for quantitative PCR using Maxima SYBR Green qPCR Master Mix (Thermo Scientific). The primer sequences for ChIP-qPCR are listed in Supplementary Table S5.

### FokI assays and image analysis

U2OS 2-6-3 cells were seeded on coverglass bottom dish (SPL) and transfected with siRNA using Lipofectamine RNAiMax (Invitrogen). After 72 h incubation, transcription was induced by treatment with doxycycline before DNA damage is induced by treatment with 4-OHT (1 μM) and Shield1 (1 μM). Cells were fixed with 4% paraformaldehyde (Sigma) and mounted with VECTASHIELD® with DAPI (Vector Laboratories). For co-immunostaining with endogenous proteins or exogenous tagged proteins, the fixed cells were further permeabilized with 0.5% triton X-100 and blocked with 1% BSA in PBS, followed by incubation with primary/secondary antibodies. Samples were imaged with Nikon A1 microscope and analyzed with the quantified fluorescence intensity at FokI-localized focus normalized with the fluorescence intensity outside of the focus.

### Cell cycle analysis

HeLa H2AX KO cells were arrested at G1 phase using single thymidine block by treatment with 20 μM thymidine for 20 h. For S phase cells, G1-arrested cells were released with the fresh medium for 4 h.

### Neutral comet assay

For neutral comet assay, U2OS cells were transfected with siH2AX(UTR) and H2AX-mutants and treated with 200 ng/ml NCS for 1 h. Cells were incubated with fresh medium after exposure of DNA damage and harvested. Cells were resuspended in PBS, mixed with LMA agarose (Trevigen) and spread over on CometSlide. The slides were incubated at 4°C and lysed with lysis buffer. Slides from lysis buffer were washed with 1× TBE buffer and immersed in 1× TBE buffer for electrophoresis at 19 V for 40 min. The slides were immersed in 70% ethanol for 5 min and dried completely at 37°C. The cells were stained using SYBR Safe DNA gel stain (Invitrogen) in TE buffer and visualized by fluorescence microscopy. Tail moment was measured by using OpenComet V1.3 software.

### DNA repair assay

DR and EJ5 cells were seeded and, next day, cells were treated with the indicated siRNA. Next day cells were electroporated with I-SceI to induce DSB and incubated for 24–48 h. After incubation, GFP positive cells were measured by FACS analysis (BD Bioscience).

### Statistical analysis

Statistics and graphs were performed using GraphPad Prism (version 5.0). Unpaired Student's *t* test was applied to compare two individual groups, while one-way ANOVA was applied to compare multiple groups. Two-way ANOVA was applied to compare multiple groups of two factors. Asterisks indicate each *P*-values (* *P* < 0.05; ** *P* < 0.01, *** *P* < 0.005).

## RESULTS

### H2A-K118 acetylation is enriched in transcriptionally active regions of the genome

**Figure 1.**
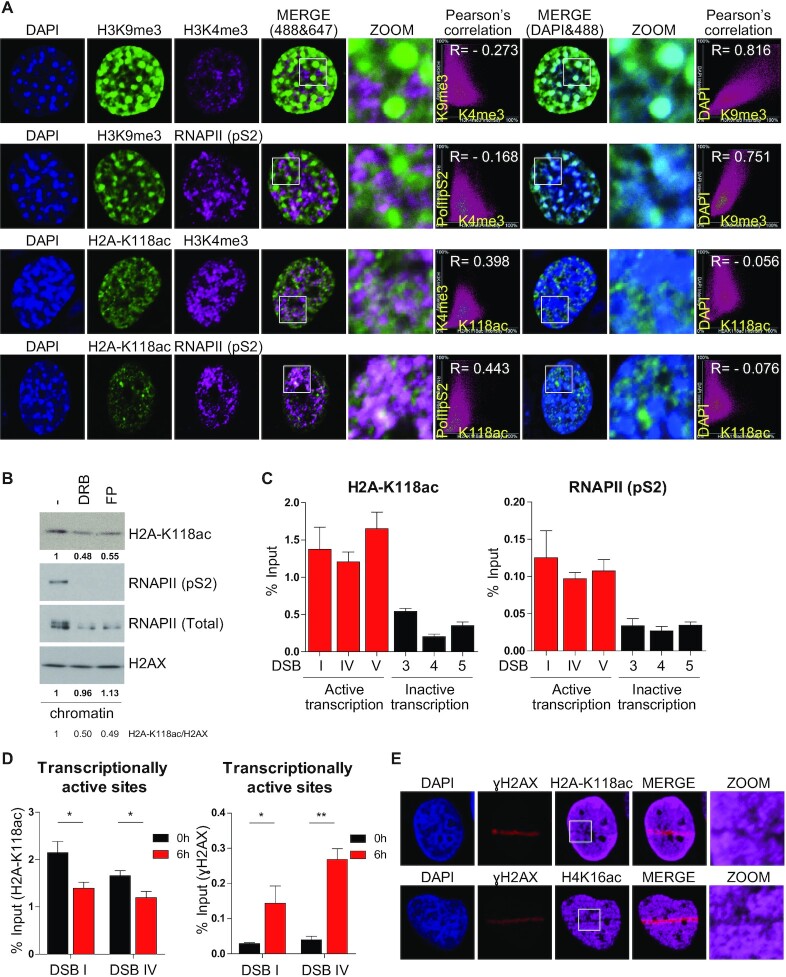
The level of H2A-K118ac is enriched at transcriptionally active site and reduced under DNA damage. (**A**) NIH3T3 cells were immunostained with the indicated antibodies and imaged under Nikon A1 confocal microscopy. H3K9me3 represents heterochromatin, while H3K4me3 and RNAPII (pS2) represent euchromatin. Pearson's correlation between H2A-K118ac and chromatin markers was analyzed. (**B**) Chromatin fractionation after treatment with DRB (100 μM) and FP (1 μM) for 2 h and immunoblotting with the indicated antibodies. (**C**) Chromatin immunoprecipitation of H2A-K118ac and RNAPII (pS2) at transcriptionally active and inactive sites in AsiSI cells in the absence of DSB. DSB I, IV and V represent transcriptionally active sites, while DSB 3, 4 and 5 represent transcriptionally inactive sites. (**D**) DIvA cells were treated with 4-OHT (2 μM) for 6 hr to induce DSBs, and ChIP was performed with H2A-K118ac at transcriptionally active (DSB I and IV) and inactive (DSB 3 and 4) sites. *P*-values were calculated using one-way ANOVA test with Bonferroni post-test. (**E**) U2OS cells were immunostained at 10 min after micro-irradiation with indicated antibodies.

Nuclear events including transcription, DNA replication, and repair occur in the context of chromatin, where the posttranslational modifications (PTMs) of histones are actively involved. We recently discovered a new histone modification of H2A acetylation at lysine 118 residue, of which status at mitotic centromeres is crucial for faithful chromosome segregation (28). We noticed that the H2A-K118ac level oscillated between interphase and mitotic phase (Supplementary Figure S1A), showing an elevation during interphase. So far, the cellular role of H2A-K118ac in interphase chromatin has not been addressed. Because transcription is turned off during mitosis, we first investigated whether H2A-K118ac was distributed throughout the euchromatin during interphase in NIH3T3 cells. Immunofluorescence staining and quantitative colocalization analysis revealed that H2A-K118ac was distributed in close to euchromatin histone markers H3K4me3 and active RNA polymerase II (phospho-Ser2). In contrast, these transcription-associated markers were distant from the heterochromatic marker H3K9me3, with a negative Pearson's coefficient for colocalization (Figure 1A). These histone markers were also well correlated with the intensity of DAPI staining, which represents heterochromatin. Using a super-resolution structured illumination microscopy (SIM), we verified that H2A-K118 acetylation was distributed in close to active transcription markers (Supplementary Figure S1B). Thus, these results suggest that H2A-K118 acetylation is enriched in transcriptionally active territories. To further test whether H2A-K118ac enrichment is correlated to transcription activities, we treated cells with DRB, an inhibitor of RNA Polymerase II (RNAPII)-mediated transcription elongation, or flavopiridol (FP), a Cdk9 inhibitor. Upon treatment with these transcription inhibitors, both RNAPII activity and H2A-K118ac levels were reduced (Figure 1B), confirming the positive correlation. Next, we examined the patterns of H2A-K118ac in the chromatin context by ChIP analysis in DIvA (DSB inducible via AsiSI) system. This cell line was originally developed to induce DSB via stably integrated AsiSI enzyme which is translocated into nucleus by treatment of tamoxifen (35–37). Later, multiple endogenous AsiSI sites in transcriptionally active (DSB I, IV, V) and inactive chromatin (DSB 3, 4, 5) have been characterized; the levels of RNAPII phosphorylated at Ser2 (pS2) are higher in the transcriptionally active regions. To investigate our hypothesis that H2A-K118ac is enriched in transcriptionally active regions, we utilize this system without inducing DSB and examined ChIP efficiency of H2A-K118ac and RNAPII (pS2) at transcriptionally active and inactive sites. In this chromatin context, the level of H2A-K118ac was also significantly elevated at transcriptionally active sites (Figure 1C). Thus, the data supported that H2A-K118 acetylation is abundant at transcriptionally active regions. Next, we addressed whether the H2A-K118ac status is changing in stressed conditions upon DNA damage. Histone H4 acetylation on lysine 16 (H4K16ac), which is associated with transcription, is reduced at the laser-microirradiated DNA lesion (38). At this time, we added tamoxifen to DIvA cells to induce DSBs throughout the genome (39). At the chromatin context, the level of H2A-K118ac at transcriptionally active sites was significantly reduced upon DSB induction, whereas γH2AX remarkably accumulated at DSB sites (Figure 1D). The basal level of H2A-K118ac was low in transcriptionally inactive sites, but a reduction in the H2A-K118ac level was also observed at the DSB 3 site (Supplementary Figure S1C). H4K16 acetylation was also slightly reduced at DSB sites in transcriptionally active chromatin (Supplementary Figure S1C). Immunostaining after micro-irradiation revealed that the levels of H2A-K118ac and H4K16ac were significantly reduced in the localized strip of DNA damage (Figure 1E). Other histone markers related to active transcription, H3K27ac and H3K4me3, were also dispersed on the damaged DNA strips (Supplementary Figure S1D). In contrast, the level of H3K27me3, which is associated with transcriptional repression, did not change at the DNA lesions (Supplementary Figure S1D). Together, these data demonstrated that H2A-K118 acetylation is enriched at transcriptionally active regions of interphase chromatin, and that its level decreases upon DNA damage.

### The chromatin remodeler RSF1 promotes DSB-induced transcriptional silencing

**Figure 2.**
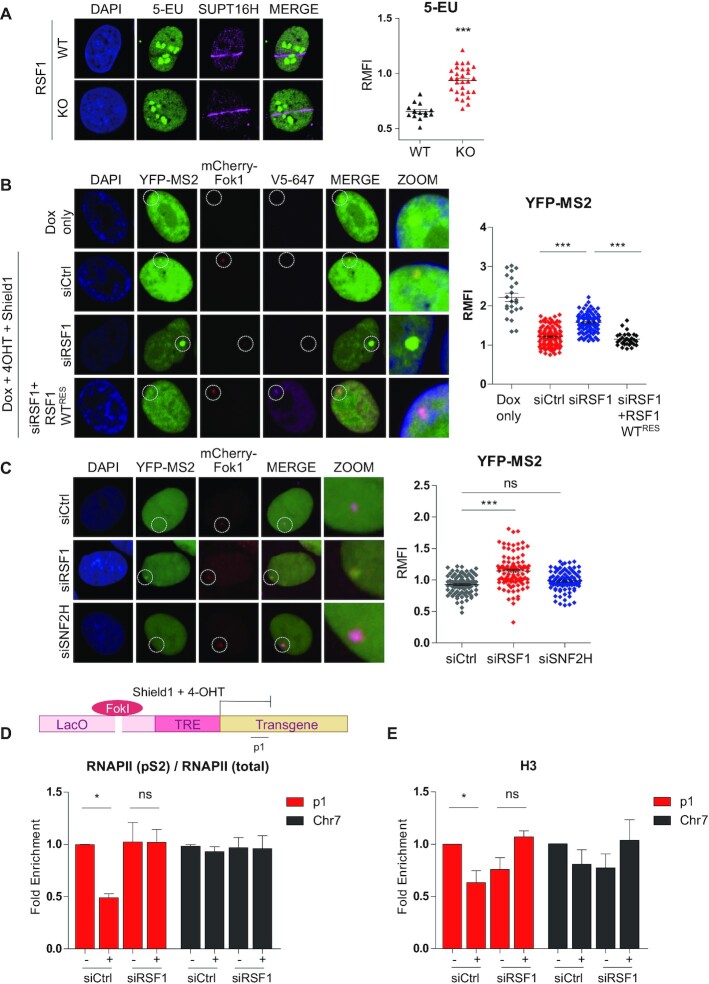
RSF1 regulates DSB-induced transcriptionally silencing. (**A**) U2OS RSF1 WT and KO cells were immunostained with 5-EU, staining nascent RNA, and SUPT16H at 40 min after micro-irradiation. Quantification of fluorescence intensity of 5-EU and γH2AX at laser strips normalized by the background fluorescence intensity was measured (Right panel). *P*-values were calculated using student's t-test. (**B**) U2OS 2-6-3 cells were transfected with siRNA and treated with doxycycline (1 μg/ml) for 1 h to induce transcription prior to treatment with 4-OHT (1 μM) and shield1 (1 μM) for 4 h to induce DNA damage. Cells were fixed and co-stained with V5-647 antibody for siRNA-resistant RSF1 WT. Quantification of fluorescence intensity of YFP-MS2 at damaged chromatin normalized by the background fluorescence intensity was measured (Right panel). P-values were calculated using one-way ANOVA test with Tukey post-test. (**C**) U2OS 2-6-3 cells were transfected with the indicated siRNAs and fixed after induction of transcription followed by DNA damage. Quantification of fluorescence intensity of YFP-MS2 at damaged chromatin normalized by the background fluorescence intensity was measured (Right panel). *P*-values were calculated using one-way ANOVA test with Tukey post-test. (**D,E**) U2OS 2-6-5 cells were transfected with siRSF1 and induced DSB for 5 h by treatment with 4-OHT and Shield1. After induction, cells were fixed and immunoprecipitated with the indicated antibodies for ChIP assay. P1 site indicates the site of DSB induced by FokI nuclease and Chr7 site indicates the site without DSB as negative control. P-values were calculated using student's t-test.

We next addressed the function of H2A-K118ac in the DDR. Previously, we showed that H2A-K118ac status is changed by the presence of RSF1, which is also necessary for an efficient DDR and DNA repair (28,30,31). Because DNA lesions have an impact on local transcription, we hypothesized that RSF1 might be involved in local transcriptional regulation through epigenetic alteration of H2A-K118ac. First, we examined the transcripts at sites of DNA damage at 40 min after micro-irradiation and subsequent immunostaining of nascent RNA with 5-ethynyluridine (5-EU) in wild-type (WT) and RSF1-knockout (KO) cells. The result revealed that transcripts were not generated at the damaged site of micro-irradiation (Figure 2A). In contrast, RSF1 depletion significantly restored 5-EU staining, indicating that RNA synthesis continued at DSB sites in RSF1 KO cells. In these cells, accumulation of SUPT16H (SPT16 Homolog), one of FACT complex that is originally co-purified proteins with RSF, was found at DNA lesions. This was consistent in cells treated with siRNA against RSF1, in which 5-EU staining was restored in the damaged strips with γH2AX accumulation (Supplementary Figure S2A). BrdU staining detecting the long stretches of ssDNA generated by DNA end resection (40) was also lacking in these cells. Thus, these results suggest that RSF1 is necessary for proper DDR as well as for DSB-induced transcriptional silencing. Although γH2AX propagation is defective in RSF1 depleted cells as we reported (30), we found that γH2AX accumulation in RSF1 KD cells was intact at early time points (10, 40 min) and its level dropped 1hr after microirradiation (Supplementary Figure S2B). Next, we used a transcription reporter system at DSBs developed by the Greenberg's lab, which is named as 2–6-3 cell line (41). The U2OS 2-6-3 cells are stably expressing mCherry-FokI endonuclease and a single DSB is induced by treatment with 4-hydroxytamoxifen (4-OHT) and Shield1. Nascent RNA transcripts are induced by doxycycline treatment at the downstream of DSB site and visualized by binding to YFP-MS2 protein. As shown in Figure 2B, YFP-MS2 accumulates at active transcription sites upon treatment with doxycycline but disappears upon induction of mCherry-FokI after treatment with 4-OHT and Shield1, which induces the DSB-induced transcriptional silencing. We measured fluorescence intensity of active transcription and showed it as RMFI (relative mean fluorescence of intensity). RMFI is the value of fluorescence intensity at transcription site divided by background signal in each individual cell. RSF1 depletion caused aberrant transcription at DSB sites, while reconstitution of siRNA-resistant RSF1 WT in RSF1-depleted cells rescued transcriptional silencing at DSB sites (Figure 2B). Together, these results clearly showed that RSF1 promotes DSB-induced transcriptional silencing at the transcribed loci.

RSF1 forms a dimer with SNF2H and the RSF complex was previously shown to regulate transcription on chromatin templates (26,27). Thus, we asked whether SNF2H is involved in transcriptional silencing at DSB sites. In contrast to RSF1 depletion, SNF2H depletion did not induce aberrant RNA transcripts (Figure 2C). Likewise, active phosphorylation of RNAPII at Ser2 was highly maintained at DSB sites in RSF1-depleted cells, but not in SNF2H-depleted cells (Supplementary Figure S2C). Thus, SNF2H is dispensable for DSB-induced transcriptional silencing, and the function of RSF1 on DSB-induced transcriptional silencing is independent to SNF2H ATPase. To explore the underlying mechanism of RSF1 on the regulation of transcription, we carried out ChIP assay at the p1 region in U2OS 2-6-5 cells (41). U2OS 2-6-5 cells are also FokI-inducible cells without stably integrated YFP-MS2. In control cells, the ratio of RNAPII (pS2)/RNAPII (total) showed a significant reduction upon DSB induction (Figure 2D) and a considerable histone H3 eviction was accompanied (Figure 2E). In contrast, the ratio of RNAPII (pS2)/RNAPII (total) in RSF1 KD cells remained unchanged and no H3 eviction was observed (Figure 2D and E). Because total RNAPII accumulation at p1 regions of DSB site remained unchanged (Supplementary Figure 2D), we concluded that RSF1-dependent transcriptional repression at DSB site might be the cause of reduced RNAPII phosphorylation (pS2) level. In addition, histone H3 eviction usually reflects open chromatin configuration and links to active transcription and thus, the observed RSF1 dependent chromatin remodeling might be linked to efficient DNA repair but not to transcriptional repression. Taken together, RSF1 depletion perturbs the DSB-induced transcriptional silencing, inducing aberrant transcription at DSB sites.

### RSF1-dependent recruitment of HDAC1 to DSB mediates the DSB-induced transcriptional repression

**Figure 3.**
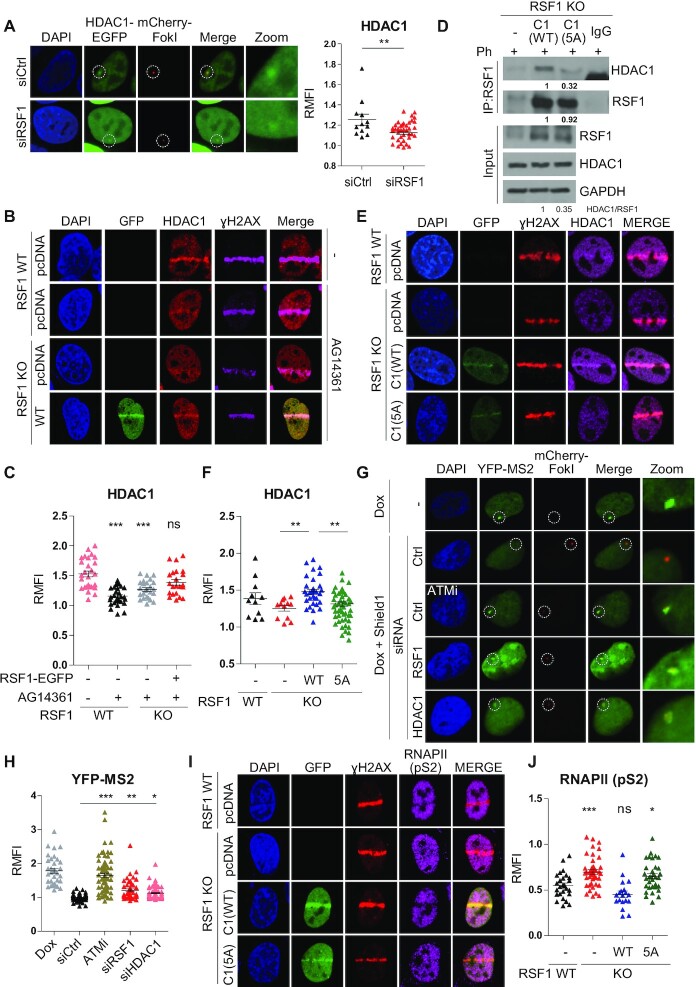
The interaction of RSF1 with HDAC1 is important for transcriptional silencing at DSB sites. (**A**) U2OS 2-6-5 cells were transfected with siRSF1 and induced DNA damage by treatment with 4-OHT and shield1 for 4 h. Localization of HDAC1-EGFP was quantified by calculating fluorescence intensity of HDAC1-EGFP at damaged chromatin normalized by the background fluorescence intensity (Right panel). *P*-values were calculated by student's t-test. (**B**) U2OS cells were immunostained with endogenous HDAC1 and γH2AX in RSF1 WT and KO cells at 10 min after micro-irradiation in the presence of AG14361, PARP inhibitor. (**C**) Relative mean of fluorescence intensity of HDAC1-EGFP at laser strip in (B) was quantified. *P*-values were calculated using one-way ANOVA test with Tukey post-test. (**D**) HeLa RSF1 KO cells were transfected with RSF1-C1 WT and -C1 5A and harvested after treatment with phleomycin for 2 h. Cells were immunoprecipitated with RSF1 antibody and immunoblotted with the indicated antibodies. Quantification of band intensities was measured by using imageJ. (**E**) U2OS cells were immunostained with endogenous HDAC1 in RSF1-C1 WT and -C1 5A transfected cells at 10 min after micro-irradiation. (**F**) Relative mean of fluorescence intensity of HDAC1 at DSB sites normalized by the background intensity was quantified. *P*-values were calculated using one-way ANOVA with Bonferroni post-test. (**G**) U2OS 2-6-5 cells were transfected with the indicated siRNA and treated with doxycycline, followed by 4-OHT and shiled1 for 4 h. Cells were fixed and imaged under confocal microscope. (**H**) Relative mean of fluorescence intensity of YFP-MS2 at DSB sites normalized by the background intensity was quantified. *P*-values were calculated using one-way ANOVA with Newman-Keuls post-test. (**I**) U2OS RSF1 WT and KO cells were harvested at 10 min after microirradiation, and RNAPII (pS2) was co-stained at sites of DNA damage after transfection of RSF1-C1 WT and -C1 5A in RSF1 KO cells. (**J**) Relative mean of fluorescence intensity of RNAPII (pS2) at DSB sites normalized by the background intensity was quantified. *P*-values were calculated using one-way ANOVA with Dunnett post-test.

We next addressed how RSF1 contributes to DSB-induced transcriptional silencing. Ingenuity Pathway Analysis (IPA) of RSF1 proteomics data (28) revealed that RSF1-interacting proteins are involved in chromatin remodeling, transcription, and DSB repair (Supplementary Figure S3A). We selected 12 of these proteins and examined whether they moved to damaged DNA lesions. Screening of RSF1-interacting proteins showed that eleven proteins except CHD1 moved to the damaged strips (Supplementary Figure S3B). SUPT16H and SSRP1, previously identified as components of the RSF-binding FACT complex (26), moved to DNA lesions, but their movements were independent of RSF1. On the other hand, EZH2, HDCA1, and the histone chaperone CHAF1A moved to DNA lesions, and depletion of RSF1 partially suppressed or abolished their recruitment to DNA lesions (Supplementary Figure S3C). Of these factors, we focused on HDAC1 because it is an important epigenetic modifier, and HDAC1 and HDAC2 promote DNA repair (38) although the underlying mechanism in the chromatin context has not been examined. First, we verified that HDAC1 was recruited to DSB sites in the FokI system. When HDAC1-EGFP was expressed in these cells, it was accumulated at DSBs, and this localization was markedly reduced in RSF1 depleted cells (Figure 3A). Likewise, live-cell imaging following micro-irradiation revealed that HDAC1-EGFP accumulated at DNA lesions, and RSF1 depletion decreased HDAC1 recruitment (Supplementary Figure S4A). On the other hand, RSF1 recruitment was not affected by depletion of HDAC1 (Supplementary Figure S4B). Previous study reported that CHD4, a component of the NuRD complex, facilitates HDAC1 recruitment at DSB sites, and CHD4 is recruited in a PARP-dependent manner (42). To exclude the effect of CHD4 on HDAC1 recruitment, we pre-treated cells with the PARP1 inhibitor AG14361 prior to micro-irradiation. Pre-treatment of U2OS RSF1 WT cells with PARP1 inhibitor decreased HDAC1 accumulation, but a small portion of HDAC1 still remained at the strip of micro-irradiation (Figure 3B). Reconstitution of RSF1 WT-GFP in RSF1 KO cells in the presence of PARP1 inhibitor caused an increase in HDAC1 recruitment (Figure 3B and C) and this might come from abundant RSF1 protein overexpressed in these cells. RSF1 accumulation is also shown to be affected by ATM kinase activity (30,31). In the presence of both AG14361 and KU55933 of ATM inhibitor, HDAC1 accumulation at the laser-microirradiated site was almost completely abrogated (Supplementary Figure S4C). Therefore, HDAC1 accumulation at DSB sites is induced by at least two independent pathways and RSF1 is an important regulator of HDAC1 recruitment at DSB sites. In co-immunoprecipitation experiments, RSF1 binds HDAC1 through LXCXE-like motif at the C-terminal region of RSF1 (28), and the substitution of these residues into alanine (RSF1-C1 5A) significantly reduced its interaction with HDAC1 under DNA damage (Figure 3D). When the RSF1-C1 wildtype and RSF1-C1 5A mutant were introduced into RSF1 KO cells, both RSF1-C1 WT and 5A moved to DSB sites upon micro-irradiation. Importantly, RSF1-C1 WT, but not RSF1-C1 5A, restored the HDAC1 recruitment to the damaged strip (Figure 3E and F). The RSF1 mutant at SQ motifs (3SA) (30) failed to accumulate at DSB sites and the subsequent accumulation of HDAC1 was also reduced (Supplementary Figure S4D). We also found that recruitment of FANCD2 or FANCI, known as downstream factors of RSF1 (31), to DNA lesions was independent to RSF1-HDAC1 complex (Supplementary Figure S4E).

We next addressed whether RSF1-dependent recruitment of HDAC1 at DSB is important for DSB-induced transcriptional repression. In FokI-coupled MS2-YFP reporter cells, *de novo* RNA transcript was detected with YFP-MS2 following doxycycline treatment (Figure 3G). Upon DSB induction, RNA transcription was no longer detected in WT cells, whereas cells depleted of either RSF1 or HDAC1 restored RNA synthesis. Quantification on YFP intensity revealed that the ATM inhibitor Ku55933 dramatically enhanced the RNA synthesis (Figure 3H) as previously observed (13). Likewise, depletion of either RSF1 or HDAC1 resulted in a significant restoration of RNA transcripts at DSBs (Figure 3G and Supplementary Figure S4F). Finally, we examined whether the RSF1-HDAC1 interaction is indeed important for the transcription activity. Under micro-irradiation active RNAPII (pS2) dissipated from the damaged strip whereas it was remained in RSF1 KO cells, consistent to the finding in Figure 1D. Again, reconstitution of RSF1-C1 WT in RSF1 KO cells, but not RSF1-C1 5A, caused a significant reduction in the level of RNAPII (pS2) on the damaged strip (Figure 3I and J), while RSF1-C1 5A still recruited at DSB sites (Supplementary Figure S4G). Together, these results indicate that the RSF1-HDAC1 interaction is important for DSB-induced transcriptional silencing.

### The RSF1-HDAC1 axis promotes the deacetylation of H2A-K118ac at DSB

**Figure 4.**
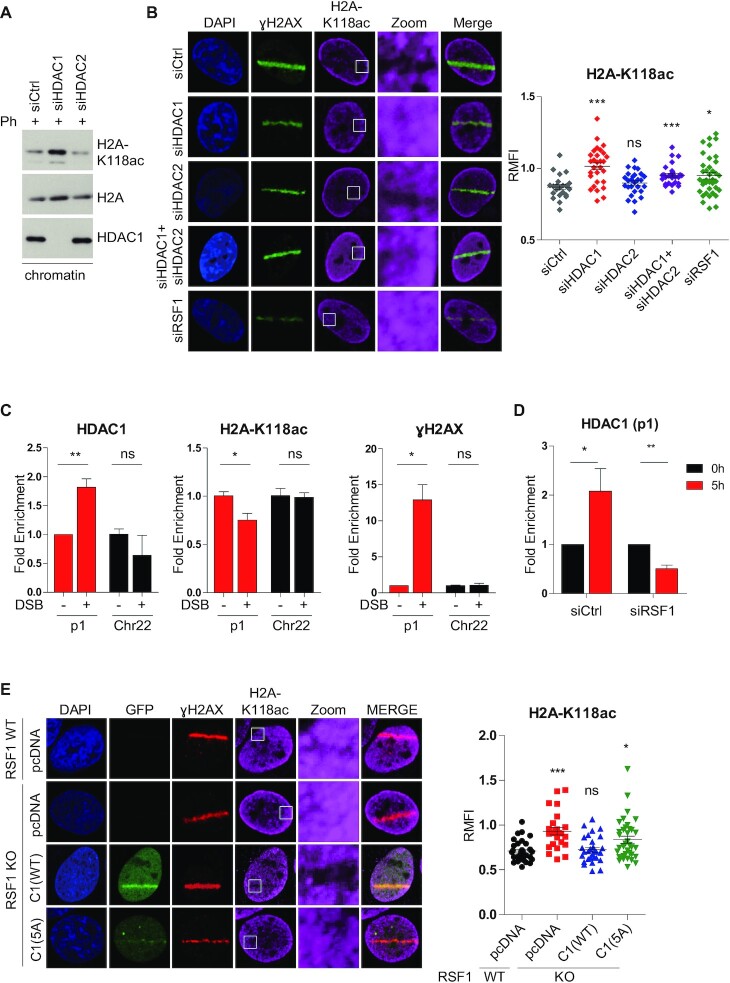
The level of H2A-K118ac was reduced at DSB sites and regulated by HDAC1. (**A**) U2OS cells were transfected with siRNA against HDAC1 or HDAC2 and chromatin was isolated after treatment with phleomycin for 2 h. (**B**) Immunofluorescence of H2A-K118ac and γH2AX at 10 min after micro-irradiation. U2OS cells were transfected with the indicated siRNA and fixed after micro-irradiation. Relative mean of fluorescence intensity of H2A-K118ac at DSB sites normalized by the background intensity was quantified (Right panel). *P*-values were calculated using one-way ANOVA with Bonferroni post-test. (**C**) Chromatin immunoprecipitation with HDAC1, H2A-K118ac and γH2AX at transcribed loci (p1) after induction of DNA damage by treatment of 4-OHT and Shield1. Fold changes of HDAC1, H2A-K118ac and γH2AX were calculated at damaged chromatin (p1) and at undamaged chromatin (Chr22). *P*-values were calculated using student's t-test. (**D**) U2OS 2–6-5 cells were transfected with siRSF1 and induced DSB for 5 h. After induction, cells were fixed and immunoprecipitated with HDAC1 at DSB sites (p1). (**E**) U2OS RSF1 WT and KO cells were transfected with pcDNA, RSF1-C1 WT, and RSF1-C1 5A and fixed at 10 min after micro-irradiation followed by immunostaining with the indicated antibodies. Relative mean of fluorescence intensity of HDAC1 at DSB sites normalized by the background intensity was quantified and graphed (Right panel). *P*-values were calculated using one-way ANOVA with Dunnett post-test.

Given that RSF1-dependent recruitment of HDAC1 to DSBs regulates transcriptional silencing, it may also induce a change in H2A-K118 acetylation status. After treatment with the radiomimetic phleomycin, the H2A-K118ac level in damaged chromatin fractions was significantly increased by siRNA targeting HDAC1, but unaffected by siRNA targeting HDAC2 (Figure 4A). Consistent with this, knockdown of HDAC1, but not HDAC2, restored the H2A-K118ac level at the strip of micro-irradiation, suggesting that HDAC1 specifically deacetylates H2A-K118. Likewise, in RSF1-knockdown cells, the micro-irradiated strip was filled with H2A-K118ac staining, and the fluorescence intensity of the strips revealed a significant increase in the H2A-K118ac level in these cells (Figure 4B and supplementary Figure S4H). The same results were obtained in RSF1 KO cells (Supplementary Figure S4I). These results strongly suggest that both RSF1 and HDAC1 are involved in deacetylation of H2A-K118ac at DSB sites. We explored this further in the chromatin context by performing ChIP analysis in FokI cells. Upon treatment with 4-OHT and Shield1, a dramatic increase of γH2AX accumulation was shown at the damaged site (p1), whereas non-damaged chromatin in chromosome 22 did not show any change. At p1 site, HDAC1 accumulated whereas the level of H2A-K118 acetylation status was reduced (Figure 4C). In this system, we verified the effect of RSF1 on HDAC1 recruitment at DSB sites and found that HDAC1 recruitment at DSB sites was indeed impaired in RSF1 KD cells (Figure 4D and Supplementary Figure S4J). Next, we examined the contribution of RSF1 to H2A-K118 deacetylation. In RSF1 KO cells, reconstitution with RSF1-C1 WT decreased the H2A-K118ac level in the micro-irradiated strip, whereas reconstitution with the RSF1-C1 5A mutant did not, suggesting that RSF1-mediated HDAC1 recruitment to the DSB is necessary for deacetylation of H2A-K118ac (Figure 4E). Together, these data elucidated that HDAC1 accumulation by RSF1 promotes the deacetylation of H2A-K118 on damaged chromatin.

### Deacetylation of H2A-K118ac promotes the efficient mono-ubiquitination of H2A-K119

**Figure 5.**
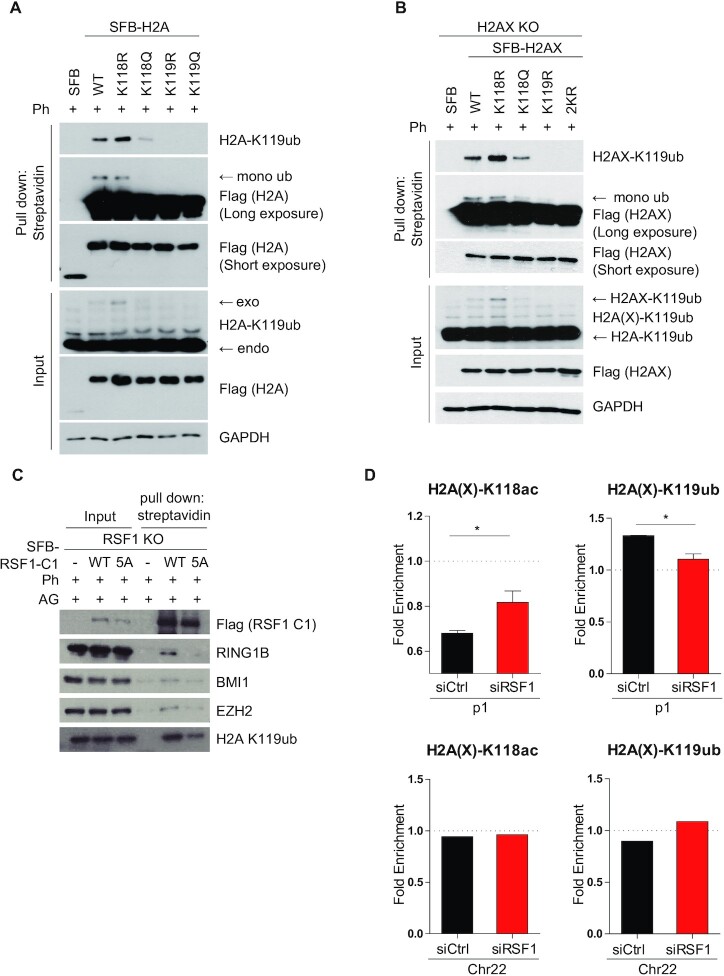
Deacetylation of H2A(X)-K118 precedes ubiquitination of H2A(X)-K119. (**A**) Various H2A mutants were transfected in U2OS cells and pulled down with streptavidin beads, followed by immunoblotting with H2A-K119 ubiquitination. (**B**) Various H2AX mutants were transfected in HeLa H2AX KO cells and pulled down with streptavidin beads, followed by immunoblotting with H2AX-K119 ubiquitination. (**C**) Streptavidin pull down assay with SFB-RSF1-C1 WT and -C1 5A after phleomycin treatment in the presence of AG14361, followed by immunoblotting with the indicated antibodies. (**D**) U2OS 2-6-5 cells were treated with siRSF1 and treated with 4-OHT and Shield1 to induce DNA damage for 5 h. Cells were lysed and performed ChIP assay with H2A(X) -K118ac and H2A(X)-K119ub after induction of DNA damage in RSF1 depleted cells. *P*-values were calculated using student's t-test.

A crucial epigenetic mark associated with DNA damage-induced transcriptional repression is mono-ubiquitination of H2A-K119 (H2A-K119ub) (13,23). A recent report (18) illuminated that H2A-K119ub indeed serves as central hub that mounts polycomb repressive machineries. Hence, we next addressed whether deacetylation of H2A-K118 by the RSF1-HDAC1 axis might affect the status of H2A-K119 ubiquitination in the context of transcriptional repression. For this purpose, we generated SFB-tagged H2A-K118 and H2A-K119 point mutants of H2A, introduced them into U2OS cells, and carried out pull-down assays after transfection of SFB-tagged H2A constructs. H2A-K118R was used as an acetylation-defective form, and H2A-K118Q as an acetylation-mimetic form. Streptavidin pull-down assay showed that mono-ubiquitination of H2A-K119 was detected upon phleomycin treatment. Intriguingly, the H2A-K119ub level in the H2A-K118R mutant showed a slight but steady increase, whereas the mono-ubiquitination of H2A-K119 was almost abolished in H2A-K118Q mutant (Figure 5A), indicating that acetylation of H2A-K118 indeed significantly alleviated the ubiquitination of H2A-K119. On the other hand, mutation of H2A-K119 to either K119R or K119Q suppressed H2A-K119 ubiquitination. Together, these results strongly suggest that H2A-K118 acetylation counteracts H2A-K119 ubiquitination. H2AX is important for DNA damage signaling and the K118 and K119 residues are well conserved in H2AX, suggesting a possibility that the H2AX-K119 residue is also ubiquitinated by RING1B/BMI1 in response to DNA damage (22,43,44). We transfected H2AX-K118 WT and its mutant forms into HeLa H2AX KO cells and found that ubiquitination of H2AX at K119 was also significantly reduced in the acetylation mimetic mutant H2AX-K118Q. Notably in this regard, acetylation-dead H2A-K118R and H2AX-K118R exhibited an increase in mono-ubiquitination at K119, compared to that of WT H2AX (Figure 5B). These results elucidated that deacetylation of H2A(X)-K118 by HDAC1 is required for efficient ubiquitination of both H2A-K119 and H2AX-K119 in response to DNA damage. Next, we further examined the effect of RSF1-mediated HDAC1 on transcriptional repression upon DNA damage by determining its interactions with components of the polycomb repressive machinery. RSF1 KO cells transfected with SFB-tagged RSF1-C1 WT and RSF1-C1 5A were treated with phleomycin along with AG14361 for 2 h to alleviate the PARP1-dependent effect on HDAC1 (Figure 3B). Pull-down assays revealed that RSF1-C1 was associated with the central components of polycomb repressive complex, RING1B, BMI1 and EZH2, of which interaction with RSF1-C1 5A were significantly reduced (Figure 5C). Consistent with this, the H2A-K119ub level was suppressed in the complex of RSF1-C1 5A. Thus, these data delineate a novel epigenetic regulation on DNA lesions: RSF1-mediated HDAC1 promotes deacetylation of H2A(X)-K118ac, which allows the polycomb complex to transfer the ubiquitin to H2A(X)-K119, thereby repressing transcription. We then verified the acetylation-ubiquitination switch at H2A(X)-K118 and -K119 in a chromatin context using ChIP assay. The reduction in the H2A(X)-K118ac level upon DNA damage (Figure 4C) was alleviated in RSF1-depleted cells, whereas H2A(X)-K119ub accumulation was lower in RSF1 siRNA cells than in RSF1-expressing cells (Figure 5D). This acetylation-ubiquitination switch was specific to the p1 site within DSB lesions, as no changes were observed on the undamaged chromosome 22 (Figure 5D). Together, these data showed that deacetylation of H2A-K118ac at DSB sites by the RSF1-HDAC1 axis promotes the ubiquitination of H2A-K119, leading to transcriptional repression.

### H2A(X)-K118 deacetylation is required for the propagation of γH2AX at DSB sites

**Figure 6.**
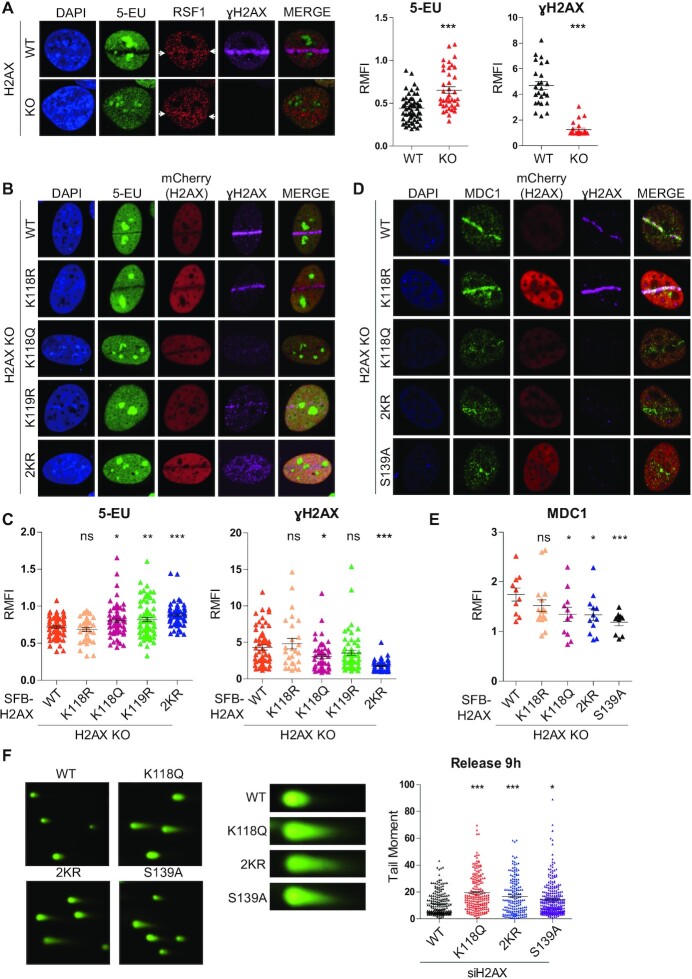
Deacetylation of H2AX-K118 is important for γH2AX propagation and DNA repair. (**A**) Nascent RNA was immunostained with 5-EU in HeLa H2AX WT and KO cells. Transcriptional repression was abolished at DSB sites in H2AX KO cells. Quantification of relative mean of fluorescence intensity was measured and graphed (Right panel). *P*-values were calculated using student's *t*-test. (**B**) H2AX KO cells were transfected with various mCherry-H2AX mutants. mCherry-positive cells were micro-irradiated and co-stained with 5-EU and γH2AX in G1 arrested cells. Cells were fixed at 40 min after micro-irradiation. (**C**) Relative mean of fluorescence intensity of 5-EU and γH2AX normalized by background fluorescence intensity was measured. *P*-values were calculated using one-way ANOVA with Dunnett post-test. (**D**) MDC1 was co-stained with γH2AX in H2AX mutant transfected cells at 40 min after micro-irradiation in G1 arrested cells. S139A mutant was used as negative control. H2AX-K118Q and H2AX-2KR mutants decreased γH2AX propagation at DSB sites after micro-irradiation. (**E**) Relative mean of fluorescence intensity of MDC1 normalized by background fluorescence intensity was measured. *P*-values were calculated using one-way ANOVA with LSD post-test. (**F**) Comet assay of H2AX-mutatnt complemented cells in H2AX KD cells after treatment with NCS for 1 h. Cells were released with fresh medium for 9 h after exposure of DNA damage and proceeded to comet assay. *P*-values were calculated using one-way ANOVA with Bonferroni post-test.

So far, we have demonstrated that RSF1 is actively involved in transcriptional silencing in proximity to the damaged chromatin through epigenetic modification on histone H2A(X). Previously, our group and others showed that RSF1 is necessary for efficient γH2AX propagation and DNA repair (30,31). Hence, we addressed whether H2A(X)-K118 deacetylation led by RSF1 might be directly linked to γH2AX propagation and DNA repair. To address this issue, we first examined whether H2AX is necessary for transcriptional silencing, in addition to its central role in DDR signaling. Interestingly, double-immunofluorescence staining for nascent RNA transcripts using 5-EU and for γH2AX revealed that neither γH2AX propagation nor transcriptional silencing occurred on the micro-irradiated strip in H2AX KO cells (Figure 6A). This observation provides the first indication that H2AX is indispensable for transcriptional silencing process at DSB sites. Next, we examined the function of H2A(X)-K118 deacetylation in γH2AX propagation. Various mCherry-tagged H2AX mutant constructs were reintroduced into H2AX KO cells and subjected to immunofluorescence staining. Both H2AX WT and H2AX-K118R induced transcriptional silencing on the damaged strips where proper γH2AX propagation occurred (Figure 6B). In contrast, the acetylation mimic H2AX-K118Q mutant promoted neither DSB-induced transcriptional silencing nor γH2AX propagation, whereas the ubiquitination-defective H2AX-K119R mutant could not induce transcriptional silencing but did not significantly affect γH2AX propagation (Figure 6B and C). Unexpectedly, the reduction of γH2AX propagation was solely observed in G1 phase, rather than in S phase (Supplementary Figure S5A). Given that the acetylation status of H2AX-K118 affected γH2AX propagation, it would affect MDC1 recruitment in G1 cells. Consistent with the patterns of γH2AX propagation, H2AX-K118Q, but not H2AX-K118R, reduced MDC1 movement to the damaged strip (Figure 6D and E). As expected, disruption of phosphorylation on the H2AX Ser139 by the S139A mutation dramatically weakened MDC1 recruitment (Figure 6D and Supplementary Figure S5B). This is the first indication that acetylation on H2AX-K118 suppresses DDR signaling. Finally, we examined the repair activity of H2AX-K118Q mutants in U2OS cells. First, we examined the level of chromatin-bound repair proteins, RPA32 and RAD51, after DNA damage and found the reduced enrichment of RPA32 and RAD51 in U2OS KO cells reconstituted with RSF1-C1 5A (Supplementary Figure S5C). We also carried out comet assay to measure the repair activity of H2AX-mutant transfected cells after exposure of DNA damaging agent. The unrepaired tails remained in H2AX depleted cells complemented with H2AX-K118Q, H2AX-2KR or H2AX-S139A whereas reconstruction of WT H2AX properly restored the damaged DNA at 9 h after phleomycin (Figure 6F and Supplementary S5D). In addition, we examined NHEJ and HR activities after complementing H2AX mutants in H2AX KD cells and found that there is a slight but consistent reduction in NHEJ repair activities in cells with H2AX-K118Q, H2AX-2KR or H2AX-S139A (Supplementary Figure S5E and F). Together, these data elucidated that deacetylation of H2A(X)-K118 is required for γH2AX propagation in addition to its central role in the DSB-induced transcriptional silencing.

## DISCUSSION

**Figure 7.**
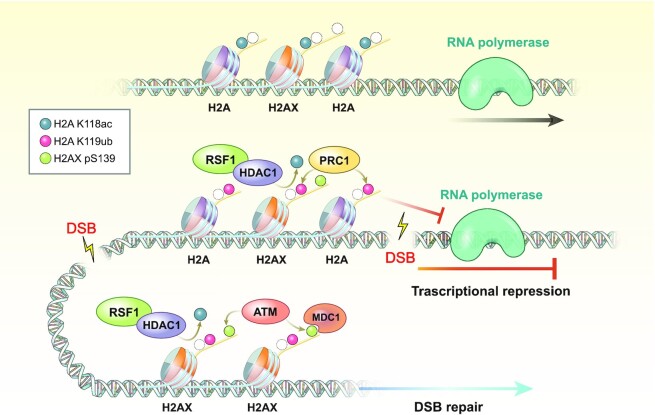
Summary. H2A-K118 is highly acetylated at transcriptionally active sites. Upon DNA damage, RSF1 recruits HDAC1 and deacetylates H2A-K118 at DSB sites, subsequently promoting ubiquitination of H2A-K119. This switch between H2A-K118ac and H2A-K119ub allows transcriptional silencing at transcribed loci and γH2AX propagation for proper DSB repair.

Here, we uncover that the RSF1 chromatin remodeler plays an epigenetic co-regulator in the DNA damage-induced transcriptional repression and DDR signaling, simultaneously. In general, histone acetylation is associated with transcriptionally active euchromatin. H4K16 acetylation marks active genes (45), in which H3K56 acetylation is also preferentially enriched (46). In response to DNA damage, the histone acetylation levels dropped (38) and however, it remained unclear whether hypo-acetylation of histones by itself is directly related to the DDR signaling. We here showed that the acetylation of H2A at K118 is enriched in transcriptionally active regions. In response to DNA damage, the RSF1-HDAC1 complex induces the deacetylation of H2A(X)-K118 and its deacetylation is indispensable for the ubiquitination of histone H2A at K119 as well as for γH2AX propagation (Figure 7). We believe that this is the first evidence that histone deacetylation in transcriptionally active chromatin generates dual signals for damage-induced transcriptional repression and DDR signaling.

How pre-existing histone modifications before DNA damage influence the DSB repair has been great interest (47). The context of chromatin at damaged locus determines DSB repair pathway choice; especially at transcribed loci, the coordinated modifications in chromatin are required to avoid the collision between DSB repair and transcription machineries (48,49). DNA-PK releases RNAPII from DNA lesion and inhibits transcription elongation at transcribed genes (50). Demethylation of histone H3K4 by KDM5A in transcriptionally active genes recruits ZMYND8-NuRD complex to DSB sites, contributing to transcription repression (51). In proliferating cells phosphorylation of H2AX-Y142 by WSTF is dephosphorylated by ATM-dependent EYA phosphatase (52) and promotes transcriptional silencing at damaged sites (53). Our work revealed that H2A-K118 acetylation, highly enriched at transcriptionally active sites, is deacetylated upon DNA damage (Figure 1). Deacetylation of H2A-K118 at transcribed locus silences the transcription proximal to DNA lesions, resolving the traffic between DNA repair and transcription machineries. Thus, deacetylation of H2A-K118 would be one of the initial signals to change chromatin landscape for transcription silencing and DDR signaling at actively transcribed loci.

Previous studies by the Greenberg group reported that ATM is an effective upstream regulator of DSB-induced transcriptional silencing. Specifically, ATM turns off the ‘transcription’ signals by dephosphorylating RNAPII at Ser2 *in cis* to DSB sites (13). Inhibition of RNAPII is linked to H2A-K119 ubiquitination (14). So far, histone H2A-K119 ubiquitination promoted by the ATM-PRC1 axis is the most well-known regulatory event associated with DSB-induced transcriptional silencing. In addition, ATM kinase phosphorylates the transcription elongation factor ENL, which contributes to H2A-K119 ubiquitination (21). We here demonstrated that deacetylation of H2A-K118 must precede H2A-K119 ubiquitination (Figure 5). Thus, acetylation of H2A-K118 in transcriptionally active regions may play an active role in suppressing the ubiquitination of neighboring histone residues on K119, thereby preventing unnecessary or faulty transcriptional repression. Similar mechanism was previously drawn in that H2A-T120 phosphorylation by VRK1 kinase inhibits ubiquitination of the adjacent K119 residue, promoting upregulation of cyclin D1 expression (54). Thus, it appeared that histone modifications next to H2A-K119 significantly affect the H2A-K119ub level. However, we cannot exclude the possibility that inhibition of H2A-K119ub following H2A-K118Q substitution might arise due to structural effect of K to Q substitution. Deacetylation of H2A-K118 is specifically mediated by HDAC1, but not by HDAC2 (Figure 4A). Previously, it is shown that HDAC1/2 in the NuRD complex HDAC1/2 are rapidly recruited to DNA damage sites and promote the global deacetylation of histones. HDAC1/2-depleted cells are hypersensitive to DNA-damaging agents, suggesting that deacetylation of histones at DNA lesions affects DNA repair (38). Here, we propose that deacetylation of histone H2A(X)-K118 by HDAC1 is specifically mediated by the chromatin remodeler RSF1. Reconstitution of RSF1 KO cells with RSF1-C1 WT, but not the RSF1-C1 5A mutant lacking HDAC1 binding, enabled interaction with PRC components (Figure 5C) and promoted deacetylation of H2A-K118 on damaged chromatin (Figure 4E). Thus, these observations demonstrate that HDAC1 associated with RSF1 promotes the deacetylation of histone H2A, which influences the chromatin landscape favorable for DNA repair and transcriptional repression.

H2A-K118 is highly conserved in the histone variant H2AX. Upon DNA damage, H2AX becomes rapidly phosphorylated at carboxy-terminal residue, Ser139, yielding so-called γH2AX. Formation of γH2AX can indicate the presence of DSBs or activated DDR signaling. Importantly, genome-wide profiling revealed that endogenous H2AX is concentrated near the transcription start site of actively transcribed genes. Accordingly, γH2AX enrichment upon irradiation also coincides with actively transcribed regions (55). Genome-wide studies also highlighted the importance of chromatin dynamics in DNA repair and transcription (12,37). Various histone modifications were screened in transcriptionally active and inactive sites to determine effects of the chromatin environment on DSB repair pathways (12,39). H3K36me3 is one of the histone modifications favorable for the resection activities in active genes (39,56). Here, we found that deacetylation of H2AX-K118 promoted γH2AX propagation specifically in G1 phase (Figure 6). The acetylation mimic H2AX-K118Q was unable to induce γH2AX propagation and MDC1 recruitment to DNA lesions (Figure 6B). Thus, deacetylation of H2AX-K118 is indeed required for phosphorylation of H2AX-S139 at DSB sites. Interestingly, although ubiquitination-defective H2AX-K119R mutant had significant effect on transcriptional silencing, it did not significantly affect γH2AX propagation (Figure 6C). Thus, the data suggest that the specificity of histone residues generate discrete signals for chromatin landscape proficient for DNA repair in G1. Furthermore, DSB at damaged active genes largely clustered in G1 and undergo repair in S phase (8,37). The effect of deacetylation of H2A-K118 on clustering the unrepaired DSBs in G1 will be the next question.

In summary, these data suggest that the RSF1, an accessory subunit of the RSF complex, is required for recruiting HDAC1 to modulate the chromatin environment and achieve efficient transcriptional silencing and DDR signaling. In this process, chromatin remodeling factors actively function in the recruiting histone modifying enzymes in addition to histone exchanges or nucleosome sliding.

## DATA AVAILABILITY

All data supporting the findings of this study are available within the article and its supplementary data.

## Supplementary Material

gkab1093_Supplemental_File
