# Supplementary material for: The chromatin remodeler RSF1 coordinates epigenetic marks for transcriptional repression and DSB repair
Source: Nucleic Acids Res. 2021 Nov 25;49(21):12268–83. doi: 10.1093/nar/gkab1093 (PMC8643642; doi:10.1093/nar/gkab1093)
Supplement: gkab1093_Supplemental_File [file gkab1093_supplemental_file.pdf]

Supplementary Figure S1

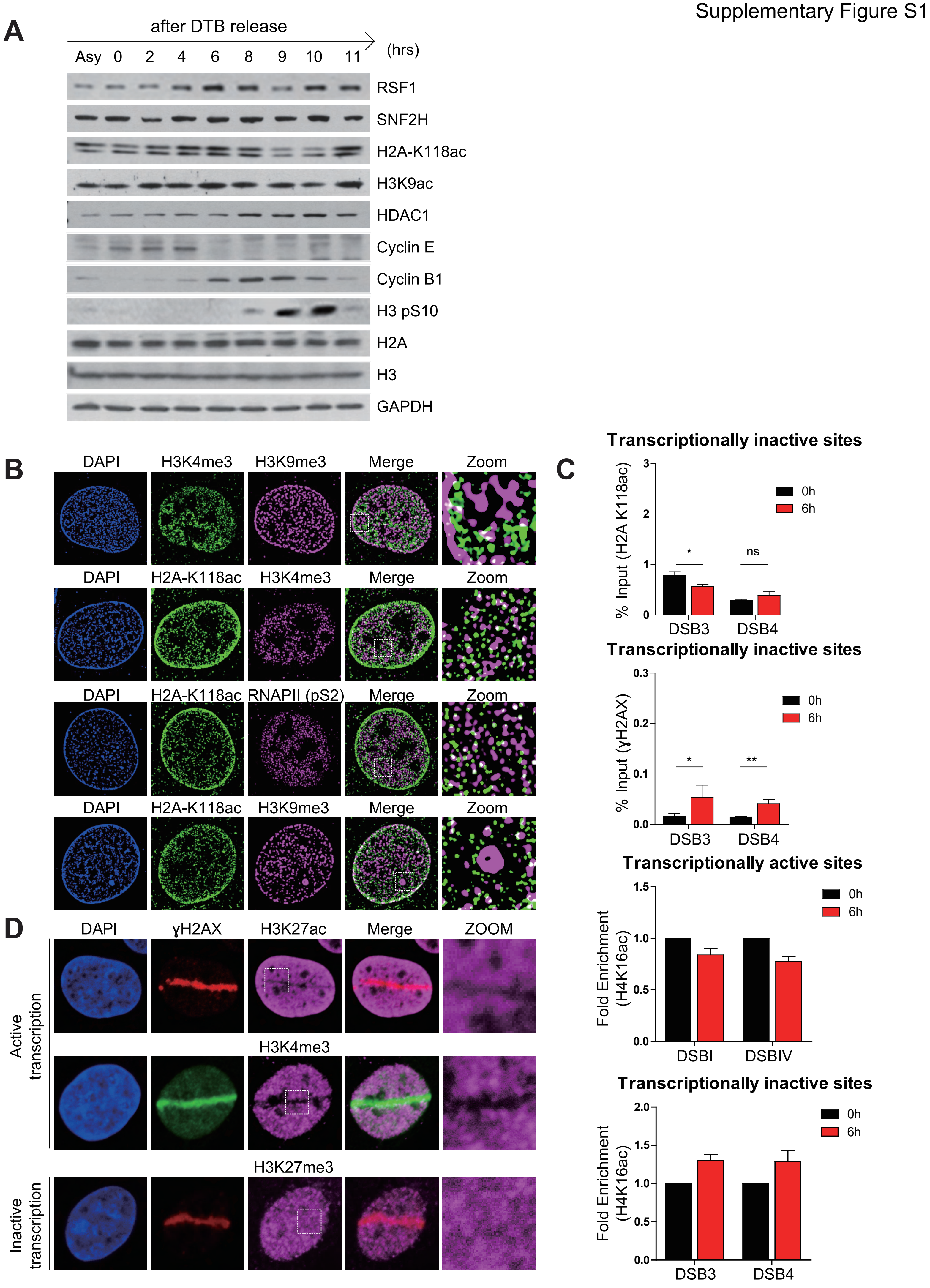

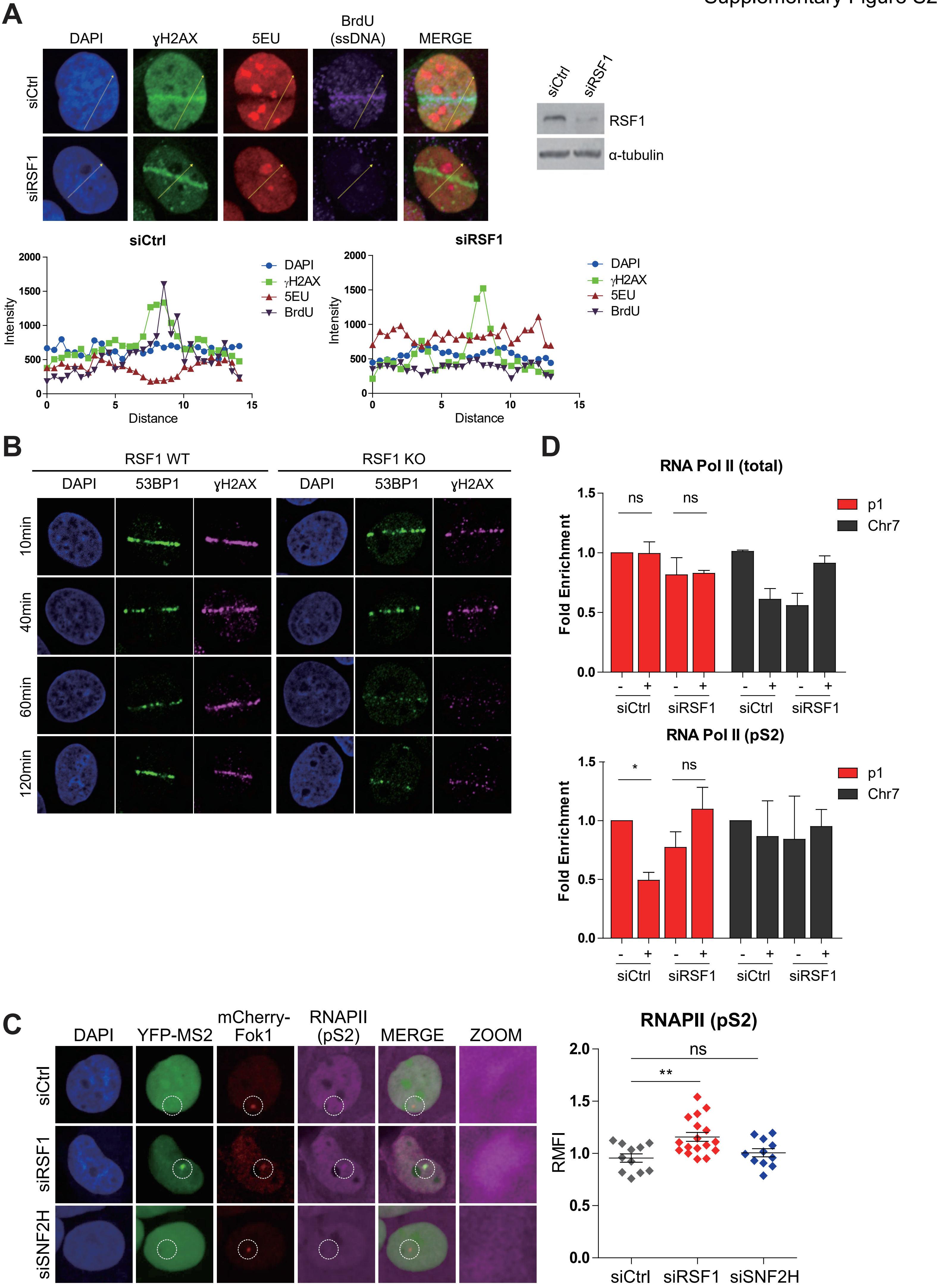
A
DAPI
γH2AX
5EU
BrdU (ssDNA)
MERGE
siCtrl
siRSF1
siCtrl
siRSF1
RSF1
α-tubulin
siCtrl
siRSF1
Intensity
Distance
DAPI
γH2AX
5EU
BrdU
B
RSF1 WT
RSF1 KO
DAPI
53BP1
γH2AX
10min
40min
60min
120min
C
DAPI
YFP-MS2
mCherry-Fok1
RNAPII (pS2)
MERGE
ZOOM
siCtrl
siRSF1
siSNF2H
RNAPII (pS2)
RMFI
ns
**
siCtrl
siRSF1
siSNF2H
D
RNA Pol II (total)
Fold Enrichment
ns
ns
p1
Chr7
siCtrl
siRSF1
siCtrl
siRSF1
RNA Pol II (pS2)
*
ns

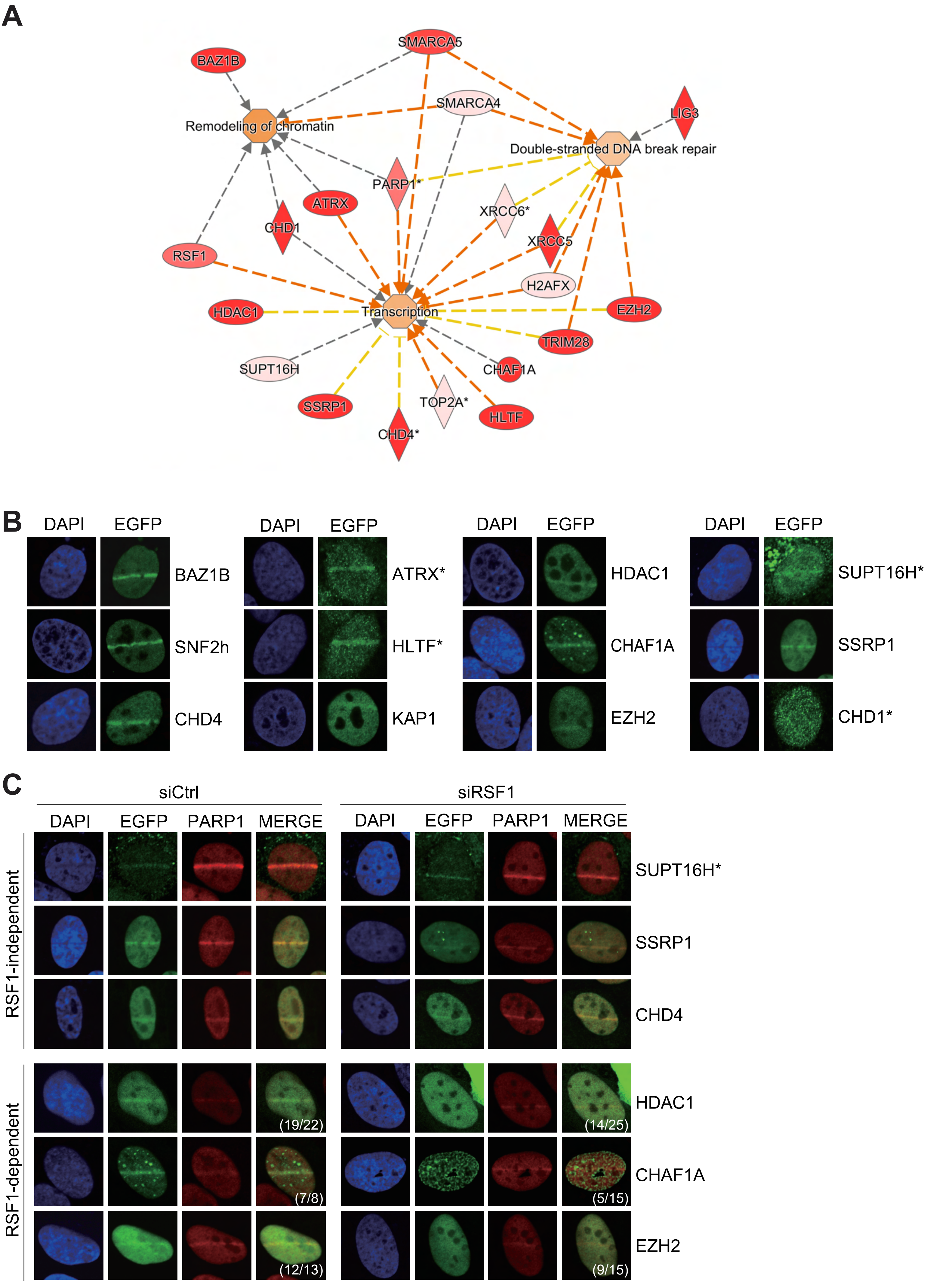
A
BAZ1B
SMARCA5
SMARCA4
LIG3
Remodeling of chromatin
Double-stranded DNA break repair
PARP1*
ATRX
CHD1
XRCC6*
XRCC5
RSF1
H2AFX
HDAC1
Transcription
EZH2
TRIM28
SUPT16H
CHAF1A
SSRP1
TOP2A*
HLTF
CHD4*
B
DAPI
EGFP
BAZ1B
SNF2h
CHD4
ATRX*
HLTF*
KAP1
HDAC1
CHAF1A
EZH2
SUPT16H*
SSRP1
CHD1*
C
siCtrl
siRSF1
DAPI
EGFP
PARP1
MERGE
RSF1-independent
RSF1-dependent
SUPT16H*
SSRP1
CHD4
HDAC1
CHAF1A
EZH2
(19/22)
(14/25)
(7/8)
(5/15)
(12/13)
(9/15)

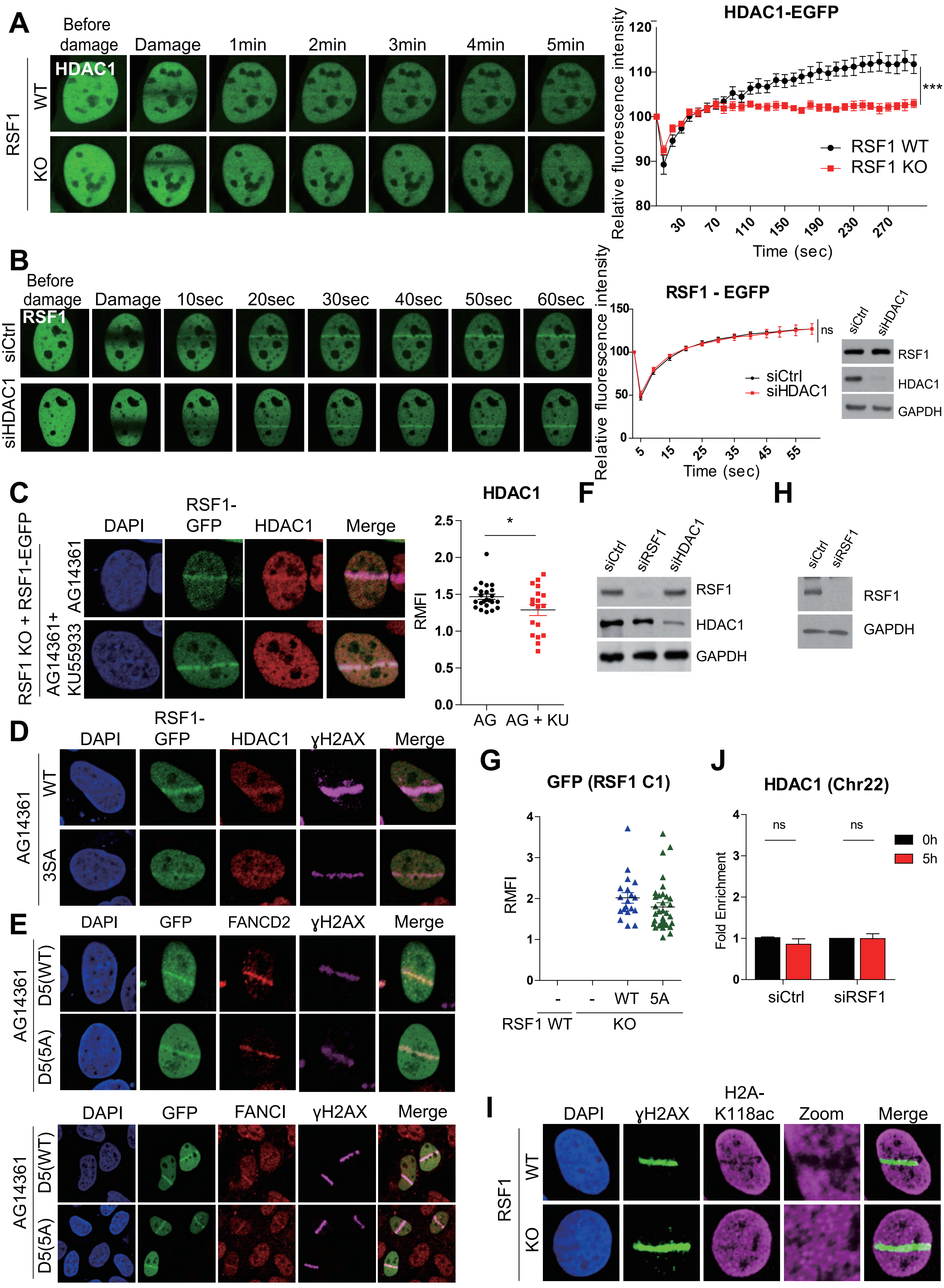
A
Before damage
Damage
1min
2min
3min
4min
5min
HDAC1
RSF1
WT
KO
HDAC1-EGFP
Relative fluorescence intensity
Time (sec)
RSF1 WT
RSF1 KO
***
B
Before damage
Damage
10sec
20sec
30sec
40sec
50sec
60sec
RSF1
siCtrl
siHDAC1
RSF1 - EGFP
ns
siCtrl
siHDAC1
RSF1
HDAC1
GAPDH
C
RSF1 KO + RSF1-EGFP
AG14361
AG14361+ KU55933
DAPI
RSF1-GFP
HDAC1
Merge
HDAC1
RMFI
*
AG
AG + KU
F
siCtrl
siRSF1
siHDAC1
RSF1
HDAC1
GAPDH
H
siCtrl
siRSF1
RSF1
GAPDH
D
AG14361
WT
3SA
DAPI
RSF1-GFP
HDAC1
γH2AX
Merge
G
GFP (RSF1 C1)
RMFI
RSF1 WT
KO
WT
5A
J
HDAC1 (Chr22)
Fold Enrichment
ns
0h
5h
siCtrl
siRSF1
E
AG14361
D5(WT)
D5(5A)
DAPI
GFP
FANCD2
γH2AX
Merge
FANCI
I
RSF1
WT
KO
DAPI
γH2AX
H2A-K118ac
Zoom
Merge

Supplemenatry Figure S5

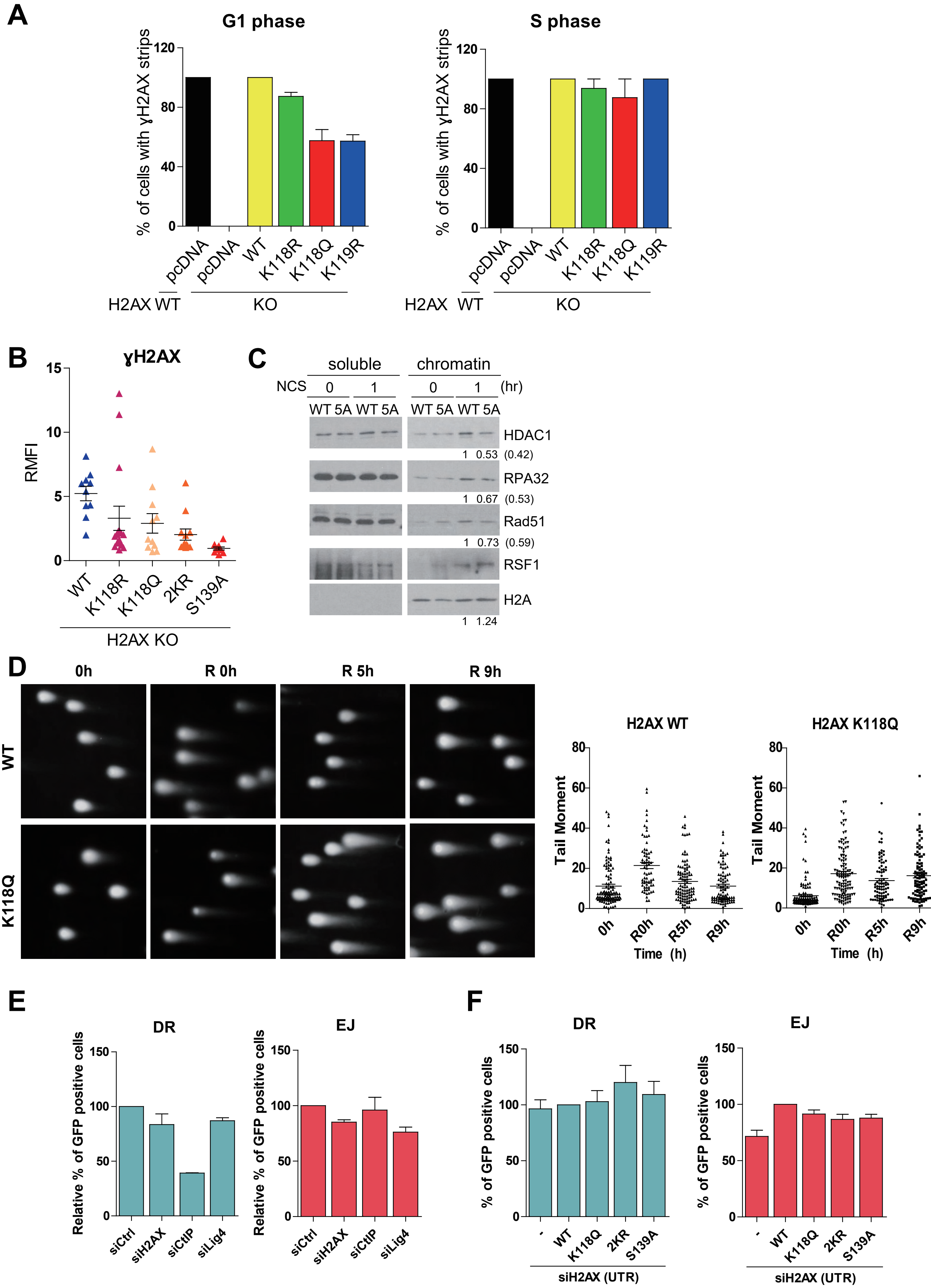

Supplementary Figure S1. H2A K118 acetylation is enriched at transcriptionally active chromatin.
(A) The level of H2A-K118ac was oscillated during cell cycle. HeLa cells were arrested at G1 phase by double thymidine block and released with fresh medium. Cells were harvested at the indicated times after release and immunoblotted with the indicated antibodies. (B) U2OS cells were immunostained with transcription markers (H3K4me3, H3K9me3, H2A-K118ac, and RNAPII (pS2)) and imaged using SIM. H2A-K118ac was located nearby active transcription markers (H3K4me3 and RNAPII (pS2)). (C) DIvA cells were immunoprecipitated with H2A-K118ac and H4K16ac for ChIP assay after induction of DSB by treatment with4-OHT for 6 hr. (D) U2OS cells were immunostained with active transcription markers (H3K27ac and H3K4me3) and inactive transcription marker (H3K27me3) at 10 min after micro-irradiation.

Supplementary Figure S2. RSF1 regulates DSB-induced transcriptional silencing.
(A) RSF1 WT and KO cells were harvested at each time point after microirradiation and immunostained with 53BP1 and γH2AX. (B) RSF1-depleted cells using siRNA were co-stained with 5-EU and BrdU under nondenaturing conditions to detect ssDNA by DNA resection at 40 min after micro-irradiation. Quantification of 5-EU, γH2AX, and BrdU at DSB sites using line scanning. (C) U2OS 2-6-3 cells were transfected with the indicated siRNAs and co-stained with RNAPII (pS2) after induction of transcription followed by DNA damage. Quantification of fluorescence intensity of RNAPII (pS2) at damaged chromatin normalized by the background fluorescence intensity was measured (Right panel). P-values were calculated using one-way ANOVA test with Tukey post-test. (D) U2OS 2-6-5 cells were transfected with siRSF1 and harvested after DSB induction and immunoprecipitated with RNAPII (total) and RNAPII (pS2) for ChIP assay.

Supplementary Figure S3. RSF1-interacting proteins related to transcription regulation are recruited at DSB sites in RSF1 dependent manner.
(A) IPA analysis of RSF1 mass spectrometry showed various proteins involved in transcription, chromatin remodeling, and double-stranded DNA break repair. (B) 12 proteins interacting with RSF1 were selected and cloned into EGFP vector. These EGFP tagged proteins of interest were transfected in U2OS cells and performed micro-irradiation. Asterisk represents the immunofluorescence of endogenous antibody against each protein, instead of EGFP tagged protein. (C) Immunofluorescence of GFP-tagged proteins in RSF1-depleted cells after micro-irradiation.

Supplementary Figure S4. RSF1 recruits HDAC1 at DSB sites and deacetylates H2A-K118ac at DSB sites. (A) Live cell imaging of HDAC1-EGFP after micro-irradiation. P-values were calculated using two-way ANOVA. (B) Live cell imaging of RSF1-EGFP after micro-irradiation. Statistical significance was calculated using two-way ANOVA. (C) U2OS RSF1 KO cells complemented with RSF1 WT were treated with AG14361 and KU55933 and microirradiated using 405nm laser. These cells were immunostained with the endogenous HDAC1 at 10 min after microirradiation and quantified its intensity at DSB sites. (D) RSF1 KO cells complemented with RSF1 WT and 3SA mutant were treated with AG14361 and immunostained with HDAC1 and ɣH2AX at 10 min after microirradiation. (E) D5(WT) and D5(5A) mutant transfected RSF1 KO cells were treated with AG14361 and induced DSB by microirradiation. Cells were harvested at 1 hr after microirradiation and immunostained with FANCD2 and FANCI. (F) Western blotting of siRSF1 and siHDAC1 in Figure 3G. (G) Quantification of RSF1-C1-EGFP in Figure 3J. Both RSF1-C1 WT and -C1 5A were properly recruited at DSB sites. (H) Western blotting of siRSF1 in Figure 4B. (I) U2OS RSF1 WT and KO cells were immunostained with H2A-K118ac at 10 min after micro-irradiation. (J) U2OS 2-6-5 cells were transfected with siRSF1 and induced DSB by treatment with 4-OHT and Shield1 for 5 hr. These cells were immunoprecipitated with HDAC1 for ChIP assay. Chr 22 represents the site without DNA damage.

Supplementary Figure S5. H2A K118 acetylation regulated by RSF1-HDAC1 is important for γH2AX propagation. (A) Quantification of γH2AX propagation at 40 min after micro-irradiation in H2AX-mutant transfected cells. H2AX-mutant transfected cells were arrested at G1 phase using

single thymidine block and released for 4 hr to undergo cells into S phase. H2AX-K118Q and -K119R showed impaired γH2AX propagation only in G1 phase, but not in S phase. (B) Relative mean of fluorescence intensity of γH2AX normalized by background fluorescence intensity was measured. (C) chromatin fractionation of RSF1 WT and 5A complemented cells after treatment with NCS for 1 hr. The samples were analyzed by western blotting with the indicated antibodies. Band intensities were measured by using imageJ and the values in bracket represents the fold reduction of each repair proteins normalized by H2A level. (D) Comet assay of H2AX-WT and –K118Q complemented cells after exposure of NCS for 1 hr. Cells were harvested at the indicated time points after releasing the cells with fresh medium. (E) DR, EJ assay with positive and negative controls for HR and NHEJ repair activities. DR and EJ5 cells were transfected with the indicated siRNAs and next day I-SceI was transfected. Cells were harvested at 24 hr after I-SceI transfection and GFP positive cells were counted by flow cytometry. (F) DR, EJ assay with the H2AX-mutant complemented cells in H2AX-KD cells. DR and EJ5 cells were transfected with siH2AX on the day before I-SceI and H2AX mutants were transfected. Cells were harvested at 24 hr after I-SceI transfection and GFP positive cells were counted by flow cytometry.

Supplementary Table 1. List of siRNAs used in this study.

Gene	Sequence	Source
Control	5’-AAU UCU CCG AAC GUG UCA CGU-3’	BIONEER
RSF1	5’-GGAAAAUGUCAAACCCAUU-3’	BIONEER
SNF2H	5’-CAG GGA AGC UCU UCG UGU UAG UGA A-3’	BIONEER
HDAC1	5’-CUA AUG AGC UUC CAU ACA A-3’	BIONEER
HDAC2	5’- GCG GAU AGC UUG UGA UGA A -3’	BIONEER

Supplementary Table 2. List of plasmids used in this study.

Plasmid	Source	Cat #
RSF1 entry clone	Harvard	HsCD00080353
HDAC1-EGFP	This paper	
RSF1-C1-WT	Sheu et al., 2008	
RSF1-EGFP	Min et al., 2014	
mCherry-H2AX	Ji et al., 2019	
GFP-WSTF	Ji et al., 2019	
SNF2H-EGFP	This paper	
GFP-CHD4	Harvard	HsCD00080095
KAP1-GFP	Dr. Aaron Goodarzi	
GFP-CHAF1A	Harvard	HsCD00339926
GFP-EZH2	Harvard	HsCD00039865
GFP-SSRP1	This paper	

Supplementary Table 3. List of mutagenesis primer sets used in this study.

Gene	Sequence	Source
RSF1-C1 5A	FW: 5’- GCGGCCGCCGCAGCGAGTGAAGAGAGC -3’<br>RV: 5’- GCTCTCTTCACTCGCTGCGGCGGCCGC -3’	iNtRON
H2A-K118R	FW: 5′- CGTACTGCTGCCCAGGAAGACGGAGAGCC-3′<br>RV: 5′- GGCTCTCCGTCT TCCTGGGCAGCAGTACG -3′	Lee et al., 2018
H2A-K118Q	FW: 5′-CCGTGCTGCTGCCTCAGAAAACTGAGAGC-3′<br>RV: 5′-GCTCTCAGTTTTCTGAGGCAGCAGACG-3′	Lee et al., 2018
H2A-K119R	FW: 5′-GCTGCTGCCTAAGAGAACTGAGAGCCACC-3′<br>RV: 5′-GGTGGCTCTCAGTTCTCTTAGGCAGCAGC-3′	Lee et al., 2018
H2A-K119Q	FW: 5′-GTGCTGCTGCCTAAGCAAACTGAGAGCCAC-3’<br>RV: 5′-GTGGCTCTCAGTTGCTTAGGCAGCAGCAC-3′	Lee et al., 2018
H2A-K118R/119R	FW: 5′- CGTACTGCTGCCCAGGCGTACGGAGAGCC-3’<br>RV: 5′-GGCTCTCCGTACGCCTGGGCAGCAGTACG-3’	iNtRON
H2AX-S139A	FW: 5’ -AAGGCCACCCAGGCCGCCCAGGAGTAC-3’<br>RV: 5’ -GTACTCCTGGGCGGCCTGGGTGGCCTT-3’	Ji et al., 2019

Supplementary Table 4. List of antibodies used in this study.

Antibodies	Source	Cat #
Rabbit polyclonal anti-Histone H3 (tri methyl K9)	Abcam	Cat# ab8898
Mouse monoclonal anti-Histone H3 (tri methyl K4)	Abcam	Cat# ab12209
Rabbit polyclonal anti-acetyl-Histone H2A (Lys118)	PTM BIO	Cat# PTM-173
Mouse monoclonal anti-RNA polymerase II RPB1 (H5)	BioLegend	Cat# 920204
Rabbit polyclonal anti-RNA polymerase II CTD repeat YSPTSPS (phospho S2)	Abcam	Cat# ab5095
Alexa Fluor 488 goat anti-mouse IgG H&L	Invitrogen	Cat# A11059
Alexa Fluor 488 goat anti-rabbit IgG H&L	Invitrogen	Cat# A11034
Alexa Fluor 546 goat anti-rabbit IgG H&L	Invitrogen	Cat# A11035
Cyanine3 goat anti-mouse IgG H&L	Invitrogen	Cat# A10521
Alexa Fluor 647 goat anti-rabbit IgG H&L	Invitrogen	Cat# A21245
Alexa Fluor 647 goat anti-mouse IgG H&L	Invitrogen	Cat# A21236
Mouse monoclonal anti-RNA polymerase II RPB1 (8WG16)	BioLegend	Cat# 664906
Mouse monoclonal anti-RNA polymerase II CTD repeat YSPTSPS antibody [8WG16]	Abcam	Cat# ab817
Rabbit polyclonal anti-Histone H2A.X (D17A3)	Cell signaling	Cat# 7631
Mouse monoclonal anti-phospho-Histone H2A.X (Ser139)	Millipore	Cat# 05-636
Rabbit polyclonal anti-phospho-Histone H2A.X (Ser139)	Novus	Cat# NB100-384
V5 tag monoclonal antibody, Alexa Fluor 647	ThermoFisher	Cat# 451098
Mouse monoclonal anti-HDAC1 (10E2)	Cell signaling	Cat# 5356
Rabbit polyclonal anti-HDAC1	Abcam	Cat# ab7028
Rabbit polyclonal anti- Histone H2A	Abcam	Cat# ab18255
Rabbit polyclonal anti-Ubiquityl-Histone H2A (Lys119) (D27C4)	Cell signaling	Cat# 8240
Mouse monoclonal anti-Anti-ubiquityl-Histone H2A Antibody, clone E6C5	Millipore	Cat# 05-678
Mouse monoclonal anti-M2 Flag	Sigma	Cat# F3165
Rabbit polyclonal anti-GAPDH	Santa cruz	Cat# sc-25778
Mouse monoclonal anti-RING1B	Active motif	Cat# 39663
Rabbit polyclonal anti-Bmi1 (D20B7)	Cell signaling	Cat# 6964
Rabbit polyclonal anti- Ezh2 (D2C9)	Cell signaling	Cat# 5246
Mouse monoclonal anti-EZH2	BD Biosciences	Cat# 612667
Mouse monoclonal anti- EZH2	Active motif	Cat# 69875
Rabbit polyclonal anti- Acetyl-Histone H3 (Lys27)	Cell signaling	Cat# 4353
Rabbit monoclonal anti-Acetyl-Histone H2A (Lys5)	Abcam	Cat# 45152
Rabbit monoclonal anti-Acetyl-Histone H3 (Lys9)	Cell signaling	Cat# 9649P
Rabbit monoclonal anti-Histone H4 (Lys 16)	Abcam	Cat# ab109463
Mouse monoclonal anti-α-Tubulin	Santa cruz	Cat# sc-23948
Mouse monoclonal anti-Histone H3 (tri methyl K27)	Abcam	Cat# ab6002
Purified mouse anti-Human PARP	BD Pharmingen	Cat# 556494
Mouse monoclonal anti-GFP(B-2)	Santa cruz	Cat# sc-9996
Rabbit polyclonal anti-MDC1	Abcam	Cat# ab11169
Rabbit polyclonal anti-53BP1	Cell signaling	Cat# 4937
Rabbit polyclonal anti-53BP1	Novus	Cat# NB100-305
Rabbit monoclonal anti-RSF1	Abcam	Cat# ab109002
Mouse monoclonal anti-CHD1	Santa cruz	Cat# sc-271626
Mouse monoclonal anti-ATRX	Santa cruz	Cat# sc-55584
Mouse monoclonal anti-HLTF	Santa cruz	Cat# sc-398357
Mouse monoclonal anti-SUPT16H (H-300)	Santa cruz	Cat# sc-28734

Supplementary Table 5. List of ChIP-qPCR primer sets used in this study.

Target	Sequence	Source
DSB I	FW: TATGGGACCAAGCGAGTAGG<br>RV: GCCTCACACACACACCCATA	Aymard et al., 2014
DSB IV	FW: GAGGAACCATTCGGACAAGA<br>RV: CTGACCAAGGAAGCCTCAAG	Aymard et al., 2014
DSB V	FW: GTCAGTATGGCCCCAGAGTC<br>RV: ACGGCTGATGGACTTAGACG	Aymard et al., 2014
DSB 3	FW: GGAAGGAGGGGCTACTAGGG<br>RV: GAAAGCCCCATTCAGTTTGA	Aymard et al., 2014
DSB 4	FW: AGGGTCGGGTTCTCTTTTGT<br>RV: CCCGGTGCTAGGAGGAAT	Aymard et al., 2014
DSB 5	FW: GGTGCCCACAGCTCTCTATG<br>RV: GAAGCCAGAGGAGTGTCCTG	Aymard et al., 2014
p1	FW: GGAAGATGTCCCTTGTATCACCAT<br>RV: TGGTTGTCAACAGAGTAGAAAGTGAA	Tang et al., 2013
Chr22	FW: CCTTCTTTCCCAGTGGTTCA<br>RV: GTGGTCTGACCCAGAGTGGT	Lacovoni et al., 2010
